# Functional Signatures of the Epiphytic Prokaryotic Microbiome of Agaves and Cacti

**DOI:** 10.3389/fmicb.2019.03044

**Published:** 2020-01-17

**Authors:** Víctor M. Flores-Núñez, Citlali Fonseca-García, Damaris Desgarennes, Emiley Eloe-Fadrosh, Tanja Woyke, Laila P. Partida-Martínez

**Affiliations:** ^1^Departamento de Ingeniería Genética, Centro de Investigación y de Estudios Avanzados del Instituto Politécnico Nacional, Irapuato, Mexico; ^2^Departamento de Biología Molecular de Plantas, Instituto de Biotecnología, Universidad Nacional Autónoma de México, Cuernavaca, Mexico; ^3^Red de Biodiversidad y Sistemática, Instituto de Ecología, Xalapa, Mexico; ^4^U.S. Department of Energy Joint Genome Institute, Walnut Creek, CA, United States

**Keywords:** arid and semiarid environments, metagenomics, phyllosphere, rhizosphere, soil, CAM plants, phototrophy

## Abstract

Microbial symbionts account for survival, development, fitness and evolution of eukaryotic hosts. These microorganisms together with their host form a biological unit known as holobiont. Recent studies have revealed that the holobiont of agaves and cacti comprises a diverse and structured microbiome, which might be important for its adaptation to drylands. Here, we investigated the functional signatures of the prokaryotic communities of the soil and the episphere, that includes the rhizosphere and phyllosphere, associated with the cultivated *Agave tequilana* and the native and sympatric *Agave salmiana, Opuntia robusta* and *Myrtillocactus geometrizans* by mining shotgun metagenomic data. Consistent with previous phylogenetic profiling, we found that Proteobacteria, Actinobacteria and Firmicutes were the main represented phyla in the episphere of agaves and cacti, and that clustering of metagenomes correlated with the plant compartment. In native plants, genes related to aerobic anoxygenic phototrophy and photosynthesis were enriched in the phyllosphere and soil, while genes coding for biofilm formation and quorum sensing were enriched in both epiphytic communities. In the episphere of cultivated *A. tequilana* fewer genes were identified, but they belonged to similar pathways than those found in native plants. *A. tequilana* showed a depletion in several genes belonging to carbon metabolism, secondary metabolite biosynthesis and xenobiotic degradation suggesting that its lower microbial diversity might be linked to functional losses. However, this species also showed an enrichment in biofilm and quorum sensing in the epiphytic compartments, and evidence for nitrogen fixation in the rhizosphere. Aerobic anoxygenic phototrophic markers were represented by Rhizobiales (*Methylobacterium*) and Rhodospirillales (*Belnapia*) in the phyllosphere, while photosystem genes were widespread in Bacillales and Cyanobacteria. Nitrogen fixation and biofilm formation genes were mostly related to Proteobacteria. These analyses support the idea of niche differentiation in the rhizosphere and phyllosphere of agaves and cacti and shed light on the potential mechanisms by which epiphytic microbial communities survive and colonize plants of arid and semiarid ecosystems. This study establishes a guideline for testing the relevance of the identified functional traits on the microbial community and the plant fitness.

## Introduction

Drylands cover 40% of the Earth’s land surface and more than two billion people depend on them (United Nations Environment Management Group, 2011; Prãvãlie, 2016). They are characterized by their low and variable amount of precipitation, high solar radiation, extreme temperatures, high potential of evaporation, high salinity and acidity, and low nutrient availability (Noy-Meir, 1973; Reynolds et al., 2007). Drylands are expected to increase up to 50–56% by the end of the 21st century as a consequence of global warming, rapid economic development, urbanization, population growth and over-exploitation of land and natural resources (Huang et al., 2016).

Agaves and cacti are plants native to the American continent. Their unique morphological, physiological and ecological features (CAM metabolism, shallow roots, spines, etc.) allow them to thrive in arid and semiarid environments (Nobel, 1988). These plants harbor a diverse microbiome that is mainly influenced by the plant compartment and their biogeography (Coleman-Derr et al., 2016; Fonseca-García et al., 2016). The dominant prokaryotic taxa in the plant-associated microbial communities are Pseudomonadales (Proteobacteria), Actinomycetales (Actinobacteria) and Bacillales (Firmicutes), while the archaeal lineage Nitrososphaera (Thaumarchaeota) is less abundant (Citlali et al., 2018). Remarkably, estimations of microbial alpha diversity in the plant-associated communities of these desert plants revealed that the rhizosphere and phyllosphere had similar prokaryotic diversity (Desgarennes et al., 2014; Coleman-Derr et al., 2016; Fonseca-García et al., 2016; Citlali et al., 2018). These results contrast with the patterns observed in other plants such as *Arabidopsis thaliana* (Bodenhausen et al., 2013), *Boechera stricta* (Wagner et al., 2016) and sugar cane (de Souza et al., 2016), in which the bacterial communities in the rhizosphere have a higher diversity than those in the phyllosphere. Importantly, the studies on agaves and cacti noted a drastic reduction of prokaryotic diversity in both the rhizosphere and phyllosphere of the cultivated *Agave tequilana* compared to the non-cultivated or native *Agave* plants (Coleman-Derr et al., 2016). This reduction is suggested to be the result of the agricultural management and/or clonal propagation, since prokaryotic diversity in the cultivated soils was similar to the one estimated in native soils (Coleman-Derr et al., 2016). However, whether this reduction in microbial diversity represents a functional loss that affects plant performance remains to be investigated.

Several bacterial strains with desirable plant-growth promotion traits (diazotrophy, indole acetic acid and siderophore production, phosphate solubilization, thermotolerance, etc.) have been isolated and characterized from the rhizosphere, root and leaf endosphere, phyllosphere and seeds of *A. tequilana*, *Agave salmiana*, *Myrtillocactus geometrizans* and *Opuntia robusta* (Desgarennes et al., 2014; Fonseca-García et al., 2016). Most of these strains also produced a diverse mixture of organic volatile compounds that promote the growth of *A. thaliana, Nicotiana benthamiana, A. tequilana* and *A. salmiana*, including novel and known compounds which biological activity was cryptic (Camarena-Pozos et al., 2019). These studies highlighted the contribution of the agave and cacti microbiome to plant fitness and the importance of studying the microbiome of non-model plants.

Epiphytic microbial communities above- and below-ground, that is the phyllosphere and rhizosphere, inhabit the interphase between the plant and the environment. These communities possess several traits that allow them to colonize and survive in the plant surfaces and promote the growth and health of their host (Vorholt, 2012; Bulgarelli et al., 2013; Vandenkoornhuyse et al., 2015; Thapa and Prasanna, 2018). However, it is uncertain if these known functions are also distributed in CAM plants adapted to arid and semiarid environments because limited research has been done in these systems. Based on the above, we would expect that non-model plants with a different ecological background would have novel functional signatures, enriching our understanding of the plant microbiome and its functions.

The main goal of this study was to dissect the characteristic functions of the epiphytic prokaryotic communities associated with different species of agaves and cacti in order to link these processes with element cycling and plant fitness, as well as to assist the design of synthetic communities for testing the ecological relevance of microbial functions in desert plants.

## Materials and Methods

### Sample Collection, Preparation, and DNA Extraction

Samples from the soil, rhizosphere and phyllosphere were taken from cultivated and natural populations of agaves and cacti in Central Mexico as previously described (Desgarennes et al., 2014; Coleman-Derr et al., 2016; Fonseca-García et al., 2016). Briefly, three healthy individuals of each plant species [cultivated *Agave tequilana* (At), wild and sympatric *Agave salmiana* (As), *Myrtillocactus geometrizans* (Mg) and *Opuntia robusta* (Or)] were sampled each in two populations at two seasons (dry and rainy) in 2012. Native and sympatric plants (As, Mg and Or) were sampled in two natural populations located in El Magueyal (Ma) and San Felipe (SF) in Guanajuato, Mexico. Plants from the cultivated *A. tequilana* (At) were sampled in two agricultural fields of two tequila companies located in Penjamo (Pe) and in Amatitan (Am) in Guanajuato and Jalisco, Mexico, respectively. Bulk soil (s) was sampled in the four sites (Ma, SF, Pe and Am) by collecting top soil (15 cm depth, 50 cm^3^, 60–65 gr) found 1 m away from sampled plants to avoid their influence. Rhizosphere (rz) and phyllosphere (e) samples were prepared by washing collected roots and leaves/stems with sterilized epiphyte buffer (50 mM KH_2_PO_4_, 50 mM K_2_HPO_4_, 0.1% Triton X-100). DNA extraction followed as previously reported (Desgarennes et al., 2014; Coleman-Derr et al., 2016; Fonseca-García et al., 2016). Since analyses using amplicon sequence data revealed that plant compartment and species/site were most important for community assembly (Coleman-Derr et al., 2016; Fonseca-García et al., 2016), shotgun metagenomic samples of the soil, rhizosphere and phyllosphere were prepared by mixing equal amounts of genomic DNA obtained from plants of the same plant species and site (six individuals render one pooled/composite sample). In total, we obtained 20 final samples: 4 from soil, 8 from the rhizosphere and 8 from the phyllosphere (4 plant species × 2 sites).

### Sequencing Data Processing

Libraries were constructed either using the KAPA-Illumina (KAPA Biosystems, Wilmington, MA, United States), or the Nextera XT kit (Illumina Inc., San Diego, CA, United States) depending on the amount of metagenomic DNA available. Sequencing was performed with an Illumina HiSeq 2500-1TB instrument using the HiSeq TruSeq SBS Sequencing kit for a 2 × 150 run. Raw reads were processed using the custom pipeline developed by the Joint Genome Institute. Quality reads were assembled using the software SPAdes v 3.12.0 (Bankevich et al., 2012) and the resulting scaffolds were annotated using the IMG Annotation Pipeline v.4.16.5 (Huntemann et al., 2016). Twenty metagenomic libraries were generated. Sequencing data is publicly available in the IMG/M database (Supplementary Table S1).

### Statistical Analysis

The general downstream analysis performed on metagenomic data is shown in Supplementary Figure S1. In-house R scripts used for each step have been deposited at github.com/vicflonun/Agaviromics/.

### Taxonomic and Functional Profiling

We estimated the copy number of each gene using the scaffold average depth. Each gene was counted for each taxonomic level (phylum, class, order and family), KO category and pathway using in-house R scripts (R Core Team, 2019). Diversity analyses were performed using the vegan package (Oksanen et al., 2019). To account for differences in library depth, gene counts were rarified (rarefy and rrarefy functions) and the taxonomic and functional alpha diversity were calculated (renyiresult function). To estimate beta diversity, we used a Non-metric multidimensional scaling analysis (NMDS) to assess all pairwise Bray–Curtis dissimilarities for the prokaryotic taxonomical and functional counts. Dissimilarity was estimated using the vegdist function of vegan package and the NMDS with the isoMDS function of the MASS package (Venables and Ripley, 2003). Abundance, diversity and NMDS were plotted using the package ggplot2 (Wickham, 2009).

### Gene Enrichment Analysis

In order to find differential abundant genes between compartments and plant species, we compared the gene counts between different groups of samples using the edgeR package (Supplementary Table S2; Robinson et al., 2010; McCarthy et al., 2012). Only genes present in more than 75% of the samples that were used in each comparison and that had an assigned function were considered for the analysis (as described in Supplementary Table S2). Then, counts were normalized (calcNormFactors function), the general dispersion was estimated (estimateGLMCommonDisp function), the gene-wise comparisons were performed (glmLRT function), and the enriched genes were selected based on their FDR (*p* < 0.05). Finally, we used the hypergeometric test (phyper function) to test for overrepresentation of each metabolic pathway in the enriched gene lists against the libraries used for comparison (Supplementary Figure S1). The differences between the soil, rhizosphere and phyllosphere were tested separately between the group of native sympatric plants and *A. tequilana* samples, since they were derived from natural and cultivated populations, respectively. The differences between plant species were tested between *A. tequilana*, *A. salmiana* and the group formed by the two cacti species, as no great differences were observed among the latter (Supplementary Table S2).

### Marker Genes Searches

We determined the taxonomic diversity and genomic context of specific differential abundant genes related to nitrogen fixation, carbon fixation, phototrophy and biofilm formation using in-house R code. We mapped these processes against the KEGG metabolic pathway database (Kanehisa and Goto, 2000). Finally, we extracted the largest gene clusters of each process and compared their genomic context against publicly available genomes from NCBI (Supplementary Table S3) and the genomes of our own bacterial strains (Supplementary Table S4) using CORASON software [Core Analysis of Syntenic Orthologs to prioritize natural products (Navarro-Muñoz et al., 2019)].

## Results

### Taxonomic and Functional Composition of the Soil, Rhizosphere, and Phyllosphere Associated With Agaves and Cacti

Our analyses revealed that the dominant bacterial phyla associated with the soil and episphere of agaves and cacti were Proteobacteria (Gamma, Alpha and Beta classes) and Actinobacteria (Supplementary Figure S2). Other abundant phyla were represented by Firmicutes, Acidobacteria, Bacteroidetes, Cyanobacteria and Gemmatimonadetes. Archaeal genes were less abundant than bacterial genes, with relative counts ranging from 0.002 to 2.3% of the total. The most abundant archaeal phyla were Euryarchaeota and Thaumarchaeota, especially the classes Nitrososphaera, Halobacteria, and Methanomicrobia (Supplementary Figure S3). Based on the KO gene counts, the proportion of metabolic processes were highly similar between metagenomes, with amino acid and carbohydrate metabolism being the most abundant processes (Supplementary Figure S4).

**FIGURE 1:**
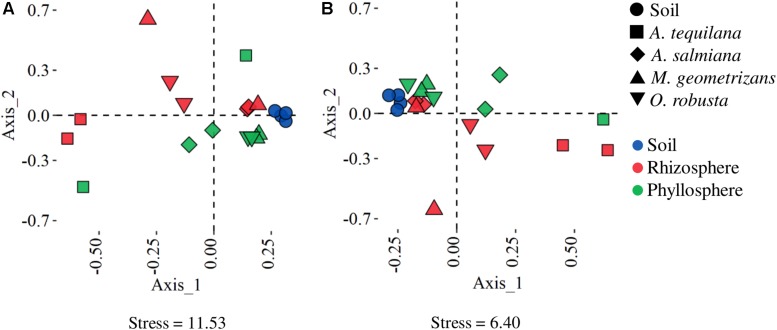
Non-metric multidimensional scaling ordination of Bray–Curtis dissimilarities between samples. The proximity between points represents the similarity between samples based on **(A)** taxonomic (family) and **(B)** functional (KO) annotation. *A. tequilana* phyllosphere from Penjamo (At.P.e) was not plotted in B due to its high dissimilarity.

The beta diversity of the metagenomes was estimated by calculating Bray–Curtis distances based on the relative abundance of KO categories and taxonomic ranks. The NMDS of gene counts at the taxonomic (Figure 1A) and functional (Figure 1B) level showed that samples tended to cluster based on the plant compartment, although the phyllospheric communities associated with *A. tequilana* were more dissimilar, especially the one derived from Penjamo (Supplementary Figure S5). The phyllosphere and rhizosphere of *A. tequilana* were less diverse than those of native *A. salmiana* and cacti (Supplementary Figure S6) and were dominated by Gammaproteobacteria, while the sample associated with the rhizosphere of *M. geometrizans* from San Felipe (Mg.Sf.rz) was dominated by Bacilli (Supplementary Figure S2). Remarkably native soils (Magueyal, San Felipe) were as diverse as the cultivated ones (Penjamo and Amatitan) (Supplementary Figure S6).

### Gene Enrichment Analysis

**FIGURE 2:**
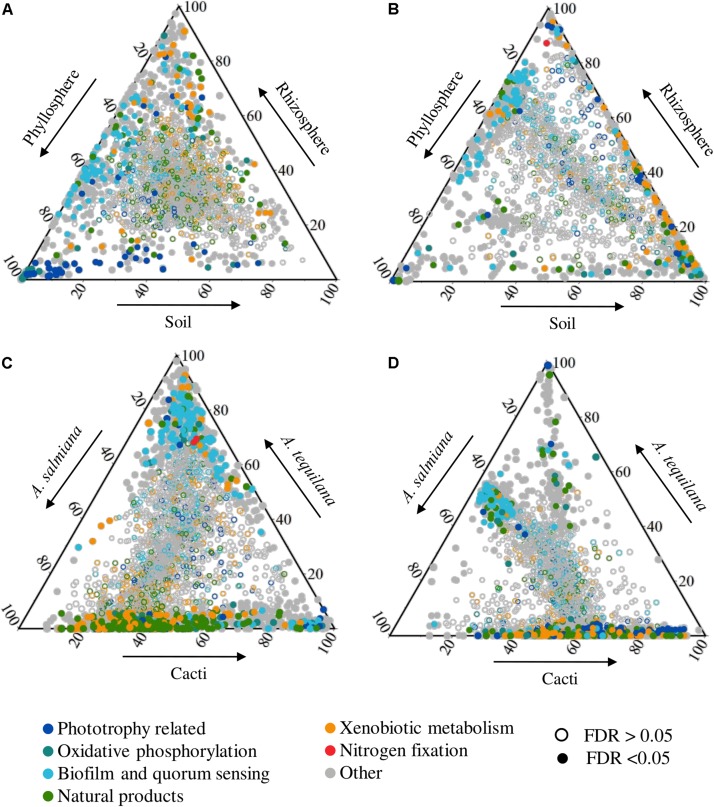
Ternary plot of differential enriched genes related to phototrophy, oxidative phosphorylation, biofilm and quorum sensing, natural products, xenobiotic metabolism and nitrogen fixation. Dots represent genes which position is defined by the ratio of its mean abundance between plant compartments (top panel) or species (bottom panel). Genes were colored based on functional groups of KO annotation and their shape is based on their FDR value in each pairwise comparison. Comparisons were made between compartments for **(A)** native sympatric plants and **(B)** *A. tequilana*, and between plants for **(C)** the rhizosphere and **(D)** phyllosphere. Arrows represent the direction of the enrichment. Phototrophy: porphyrin and chlorophyll metabolism [ko00860], carotenoid biosynthesis [ko00906], photosynthesis [ko00195] and puf/puh/puc genes. Oxidative phosphorylation: oxidative phosphorylation [ko00190]. Biofilm and quorum sensing: quorum sensing [ko02024], biofilm formation-*Pseudomonas aeruginosa* [ko02025], biofilm formation-*Escherichia coli* [ko02026], flagellar assembly [ko02040], biofilm formation-*Vibrio cholerae* [ko05111], bacterial chemotaxis [ko02030]. Natural products: metabolism of terpenoids and polyketides, biosynthesis of other secondary metabolites KEGG pathways. Xenobiotic metabolism: xenobiotics biodegradation and metabolism KEGG pathways. Nitrogen fixation: nif genes.

In order to find differential abundant genes between plant compartments and species, we performed a gene enrichment analysis using the edgeR package (Figure 2 and Supplementary Table S2). We also tested for overrepresentation of metabolic pathways in the list of enriched genes (Supplementary Figure S7). We decided to look for compartment specific differences in the group of native sympatric plants (Figure 2A) separately from the cultivated *A. tequilana* samples (Figure 2B), since they were derived from natural and cultivated populations, respectively. We also looked for species-specific gene enrichments between *A. tequilana*, *A. salmiana* and cacti, as no great differences were observed among cacti species alone (Supplementary Table S2), in both the rhizosphere (Figure 2C) and the phyllosphere (Figure 2D). Archaeal genes did not contribute to the enrichment of genes in most of the comparisons performed.

### The Phyllosphere of Agaves and Cacti

Most of the enriched genes of the phyllosphere compared to the rhizosphere in native plants (Figure 2A) suggested a phototrophic lifestyle, where *puf* reaction centers, cyanobacterial photosystems, electron chain genes, carotenoid, chlorophyll and bacteriochlorophyll biosynthetic genes were enriched with some of these pathways overrepresented (Supplementary Figure S7). Furthermore, genes for the transport of different carbon sources [xylobiose, chitobiose, inositol and bicarbonate (1 of 4)] and the excretion of capsular polysaccharides were enriched (Supplementary File S1, Transporters). When compared to the native soils, the phyllosphere was enriched and overrepresented in genes coding for the formation of biofilm and quorum sensing (e.g., acyl homoserine lactone synthase, Figure 2A, Supplementary File S1, Biofilm), secretion systems and several transporters (Supplementary Figure S8). The most significant were for the autoinducer-1, capsular polysaccharides, glutathione, histidine, inositol, manganese/iron, maltose, ascorbate, cellobiose, galactitol, methyl galactoside, among others. In both comparisons (vs. soil and vs. rhizosphere), the methanol dehydrogenase (mhd1) gene was also enriched in the phyllosphere (Supplementary File S1, Carbon metabolism).

The phyllosphere of *A. tequilana* had few enriched genes compared to its rhizosphere (Figure 2B and Supplementary Table S2) suggesting functionally homogeneous epiphytic communities compared to native plants (Figure 1 and Supplementary Table S2). The phyllosphere of *A. tequilana* was enriched and overrepresented in genes related to the metabolism of aromatic amino acids and purines, ribosomal proteins, together with glutamine and cellobiose transporters, compared to the rhizosphere, but not phototrophy related genes as in the phyllosphere of native plants (Supplementary File S1, Phototrophy and Supplementary Figure S7). Also, when compared to soils, biofilm formation and quorum sensing (Figure 2B), methane metabolism and different ABC and PTS transporters were enriched, similarly to what was observed in native plants (Supplementary File S1).

By comparing the phyllospheric communities between plant species, it became clear that native and sympatric plants were functionally similar, thus we retrieved few differential genes (Figure 2D and Supplementary Table S2). When compared with *A. tequilana*, these plants were enriched (*A. tequilana* reduced) in several genes coding for the degradation of different xenobiotics, carbohydrate metabolism and transport, amino sugar metabolism, carbon metabolism (Supplementary File S1 and Supplementary Figure S7), carbon fixation, and different secondary metabolite pathways (Figure 2D and Supplementary File S1). *A. tequilana* had few enriched genes compared to *A. salmiana*, mainly belonging to the amino acid metabolism (Supplementary Figure S8). Interestingly both *Agave* species where enriched in quorum sensing and biofilm formation genes compared with cacti (Figure 2D).

### The Rhizosphere of Agaves and Cacti

When compared to the phyllosphere, the rhizospheric communities of native plants were significantly overrepresented in the metabolism of cysteine and methionine (Supplementary Figure S7), enriched in the metabolism of carbon, different amino acids and nitrogenous bases (Supplementary File S1). The most significant transporters were for example lactose/l-arabinose, taurine, aldouronate, glutamine, octopine/nopaline, dipeptide transport systems, D-methionine, cysteine and sodium (Supplementary File S1). Interestingly, 90% of biofilm, chemotaxis and flagellar assembly and quorum sensing genes were enriched in both the phyllosphere and rhizosphere (Figure 2A) compared to the soils, such as the adenylate cyclase, and S-ribosylhomocysteine lyase and poly-N-acetyl glucosamine (PGA) biosynthesis (Supplementary Figure S11 and Supplementary File S1). These findings suggest the importance of biofilm formation as a core trait in the microbial-plant and microbial-microbial interactions in these desert plants.

As mentioned above, the rhizospheric prokaryotic communities of *A. tequilana* had very few differential genes when compared to its phyllosphere (Figure 2B). These include transporters for sorbitol, erythritol, octopine/nopaline iron and the heme group. When compared to cultivated soils, the *A. tequilana* rhizosphere was enriched in genes of nitrogenous base, glycerophospholipids metabolism (Supplementary File S1) and the nifH gene, but also in other genes enriched in the rhizosphere of sympatric plants such as biofilm formation, chemotaxis, quorum sensing and other processes (Figure 2B).

The rhizospheres were also very similar among sympatric plants with few differential genes (Figure 2C). Native plants were enriched and overrepresented (*A. tequilana* reduced) in several genes of carbohydrate and carbon metabolism, amino acids, xenobiotic metabolism and the biosynthesis of several natural products (Supplementary File S1 and Supplementary Figure S7). Interestingly, the rhizosphere of *A. tequilana* was mainly enriched in genes related to biofilm formation and quorum sensing when compared to native plants (Figure 2D). The divergence in the rhizosphere of *A. tequilana* might be linked to the dominance of *Enterobacteriales* and the low abundance of Actinobacteria and other proteobacterial taxa (Supplementary Figure S2).

### The Soils of Agaves and Cacti

Native and cultivated soils were very similar between them, since they had very few differential genes (Supplementary Table S2). Native soil microbial communities were similar to the phyllosphere and rhizosphere of native plants with few enriched genes (Figure 2A and Supplementary Table S2). These results lend further support to the notion that the soil is a pool of microorganisms for other plant compartments. Compared to the rhizosphere, soil communities were enriched in genes related to phototrophy, similarly as their presence in the phyllosphere.

Soil communities of *A. tequilana* fields were more dissimilar to its rhizosphere and phyllosphere, with several differential abundant genes (Supplementary Table S2). Compared to the phyllosphere, they were enriched in genes of transport and signaling, the metabolisms of propanoate, butanoate, glyoxylate, amino sugars, nucleotides, fructose and amino acids (Supplementary File S1 and Supplementary Figure S7). Compared to the rhizosphere, they were enriched in genes of similar processes, but also in the methane metabolism and carbon fixation (Supplementary Figure S7).

### Linking Genes to Community Composition and Functionality

#### Phototrophy

**FIGURE 3:**
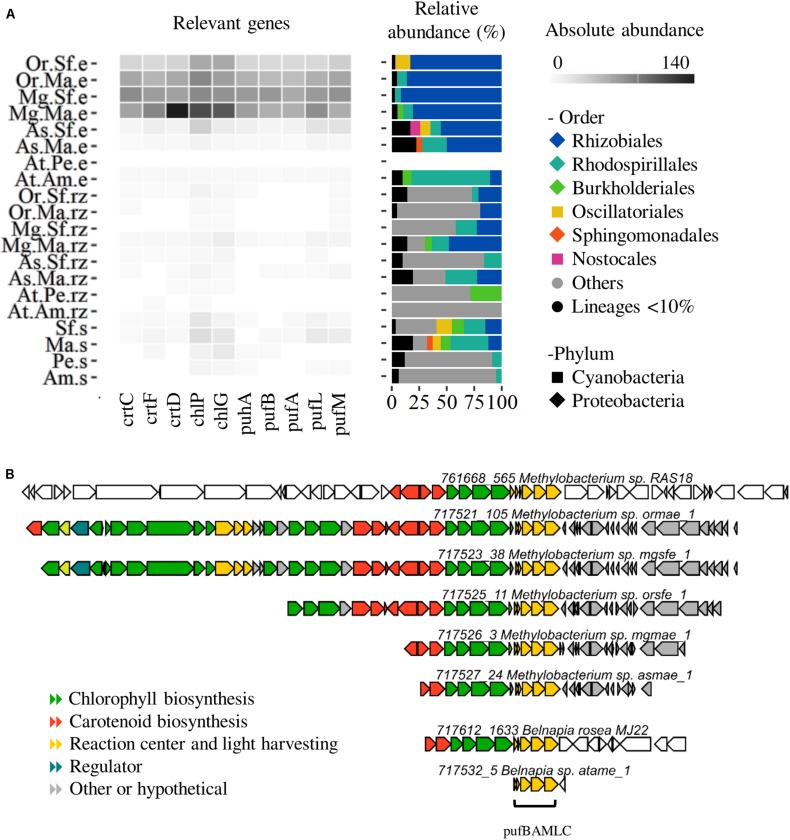
Aerobic anoxygenic phototrophs (AAP) profiling. **(A)** Abundance and taxonomic profile of phyllosphere enriched genes (pufABLM, puhA), bacteriochlorophyll biosynthesis genes (chlPG) and carotene biosynthesis genes (crtCDF). **(B)** AAP clusters of *Methylobacterium* and *Belnapia* scaffolds. Species: At, *A. tequilana*; As, *A. salmiana*; Mg, *M. geometrizans*; Or *O. robusta*. Sites: Am, Amatitán; Pe, Pénjamo; Ma, El Magueyal; Sf, San Felipe. Compartment: s, bulk soil; rz, rhizosphere; e, phyllosphere. Others: taxa not present in the phyllosphere. Lineages < 10% – low abundant taxa.

Several genes related to phototrophy were enriched in the phyllosphere of sympatric and native plants compared to the rhizosphere (Figure 3A). These genes included the biosynthesis of chlorophyll and bacteriochlorophyll *a* and *b* from protoporphyrin IX; genes from several steps of the synthesis of carotenes (lycopene and beta carotene) from Geranyl-Geranyl-PP, especially the ctrCDF orthologous related to the biosynthesis of spirilloxanthin, TH – spirilloxanthin and spheroidene [the main carotenes in aerobic anoxygenic phototrophs (AAP)] (Zheng et al., 2011). Finally, the reaction center pufABLM of AAP bacteria and the main subunits of the cyanobacterial photosystems I and II were also enriched (PSI and PSII, Supplementary File S1).

In the phyllosphere of CAM plants, AAP marker genes were almost completely related to Alphaproteobacteria (Figure 3A). In *A. salmiana* and cacti, reaction centers and pigment genes were dominated by Rhizobiales (Methylobacteriaceae, ∼90%), while very few belonged to Rhodospirillales, Sphingomonadales and Burkholderiales. In contrast, the *A. tequilana* phyllosphere (Amatitan) was mainly assigned to Rhodospirillales (Acetobacteraceae).

In native plants, the longest pufABLM gene cluster was related to *Methylobacterium* while in *A. tequilana* to *Belnapia* (Figure 3B). The metagenome retrieved AAP gene clusters were compared to 99 public *Methylobacterium* and *Belnapia* genomes using CORASON (Supplementary Figure S9A). The phyllospheric *Methylobacterium* clusters included mainly the reaction center, chlorophyll and carotenoid biosynthetic genes (chlDIO-crtIBCD-GGPS-crtF-bchCXYZ-pufBAMLC). This pattern was similar across *Methylobacterium* genomes, but slightly different to *Methylobacterium* RAS18, a methylotrophic strain isolated from *A. salmiana* (Supplementary Figure S9A and Supplementary Table S5). The small pufABLMC of *Belnapia* (At.Am.e sample) was similar to other genomes including our phyllospheric strain *Belnapia rosea* MJ22 (Supplementary Figure S9B).

We found scaffolds containing the enriched photosystem genes (psa, psb, pet) from diverse cyanobacterial clades (Chroococcidiopsidales, Synecoccocales, Nostocales, Oscillatoriales, Chroococcales, Supplementary Figure S10A) and we have already isolated two strains, *Nodosilinea* sp. and *Leptolyngbya* sp. (Synecoccocales) from the phyllosphere of *A. tequilana* (Supplementary Figure S10B and Supplementary Table S5). However, several of these genes were also assigned to non-photosynthetic lineages, such as Bacillales and Lactobacillales, but also *Staphylococcus* and *Trichococcus* (Supplementary Figure S10A).

#### Nitrogen Fixation

**FIGURE 4:**
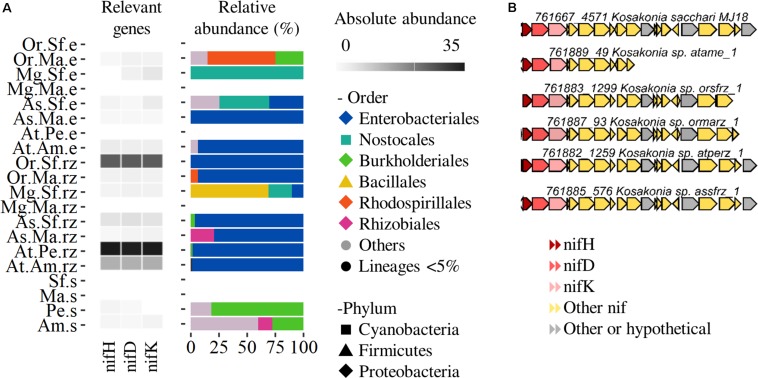
Nitrogen fixation profiling. **(A)** Abundance and taxonomic profile of the differential enriched nifHDK genes. **(B)** Nitrogen fixation clusters of Kosakonia/*Enterobacter* scaffolds. Species: At, *A. tequilana*; As, *A. salmiana*; Mg, *M. geometrizans* Or *O. robusta*. Sites: Am, Amatitán; Pe, Pénjamo; Ma, El Magueyal; Sf, San Felipe. Compartment: s, bulk soil; rz, rhizosphere; e, phyllosphere. Others: taxa not present in the rhizosphere. Lineages < 5% – low abundant taxa.

For agaves and cacti, nitrogen represents the most limiting macro-nutrient (Nobel, 1988) and our previous work reported on the presence of diazotrophs in the bacterial communities associated with agaves and cacti (Desgarennes et al., 2014; Fonseca-García et al., 2016). We analyzed the genes involved in nitrogen fixation. The nif genes were not abundant, but were found across all the plant compartments and species with the exception of the sample derived from the phyllosphere of *A. tequilana* in Penjamo (At.P.e). Only the nifH gene was enriched in the rhizosphere of *A. tequilana* compared to the cultivated soils and the rhizosphere of native plants (Figures 2B,C). In the rhizosphere of both agaves and cacti the most retrieved clusters (>3 genes) belonged mostly to *Enterobacteriales* such as Kosakonia/*Enterobacter*, but included also Nostocales, Rhodospirillales, Bacillales, and Burkholderiales (Figure 4A). These clusters were similar to the one identified in the diazotrophic strain *Kosakonia sacchari* MJ18 isolated from the root endosphere of *A. salmiana* (Figure 4B).

#### Biofilm Formation

**FIGURE 5:**
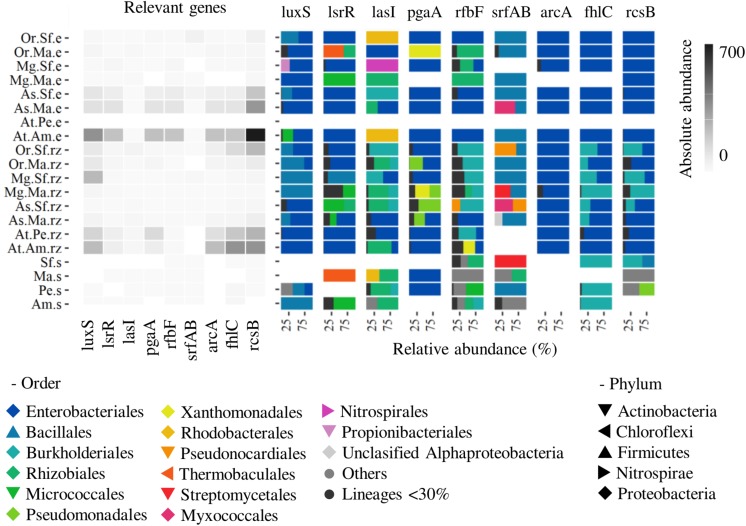
Biofilm formation profiling. Abundance and taxonomic profile of quorum sensing genes (luxS, lasI, lsr), rhamnolipids (rfbF), PGA (pgaA) and surfactin (srfAB) biosynthesis, regulators (arcA, fhlC, rscB). Species: At, *A. tequilana*; As, *A. salmiana*; Mg, *M. geometrizans*; Or, *O. robusta*. Sites: Am, Amatitán; Pe, Pénjamo; Ma, El Magueyal; Sf, San Felipe. Compartment: s, bulk soil; rz, rhizosphere; e, phyllosphere. Others: taxa not present in the rhizosphere or phyllosphere. Lineages < 30% – low abundant taxa.

Biofilm formation was enriched and overrepresented in the epiphytic microbial communities of agaves and cacti (Figure 5). Biofilm formation is regulated by quorum sensing signaling and related to other processes like stress response, flagellar assembly and chemotaxis. The main metagenomic signatures were the genes luxS (S-ribosylhomocysteine lyase) and lsrR (Transcription factor for AI-2 transporter and kinese) involved in quorum sensing in Gram negatives and positives, but also the gen lasI (acyl homoserine lactone synthase) related to the quorum sensing via AI-1 in Gram negatives. Genes related to the formation of the matrix such as pgaAB for the biosynthesis of poly-N-acetyl glucosamine (PGA), rhlC for the biosynthesis of rhamnolipids and srfAB for the biosynthesis of surfactins were also enriched and overrepresented, similarly as some response regulators like arcAB (energy and oxygen), flhCD (flagellar assembly), rcsBC (envelope), and PTS-glu/cyaA, among others (Supplementary File S1). Biofilm formation regulator genes were enriched in the epiphytic compartments compared to soils (especially in the sympatric plants), but when compared by plant species, it was clear that they were most abundant in the phyllosphere of both agaves compared to cacti, and in the rhizosphere of *A. tequilana* compared to native plants (Figure 5 and Supplementary File S1). *Enterobacteriales* were responsible for the enrichment of most of the former genes especially in the phyllosphere (Figure 5). Autoinducer 2 associated genes were mostly related to *Enterobacteriales* and Bacillales. LasI was mainly related to Rhizobiales, *Enterobacteriales* and Burkholderiales. The pgaAB genes that are important for the PGA production in *Escherichia coli* were assigned almost completely to *Enterobacteriales* in the phyllosphere, but also to Pseudomonadales and Xanthomonadales in the rhizosphere. The regulator genes were not only represented by *Enterobacteriales*, but also by Bacillales (PTS-Glu), Burkholderiales (rcsBC, flhCD) and Pseudomonadales (cyaA) in the rhizosphere. The gene rfbF (rhamnolipids) was assigned to *Enterobacteriales* and Rhizobiales in the phyllosphere and to other diverse lineages in the rhizosphere, while the sfrAB for surfactin production was assigned mainly to Bacillales (Figure 5 and Supplementary Figure S12).

## Discussion

### Phototrophy Is a Signature Trait of the Phyllosphere of Agaves and Cacti

Photosynthesis related genes were enriched in the metagenomes of the phyllosphere, a finding which correlated with our previous work were cyanobacterial OTUs (mainly *Microcoleus* genus) were abundant (2–16%) in the phyllosphere of agaves and cacti (Citlali et al., 2018). Cyanobacteria might not be common to the phyllosphere of all land plants. They have been reported in the phyllosphere of mangrove trees (Rigonato et al., 2012), tropical rainforest vegetation (Fürnkranz et al., 2008; Kim et al., 2012), rice (Venkatachalam et al., 2016; Thapa and Prasanna, 2018) and grapevine (Singh et al., 2018), but not in the leaf microbiome of *Arabidopsis* (Bodenhausen et al., 2013), bean, canola (Copeland et al., 2015) or sugar cane (de Souza et al., 2016). Cyanobacteria might be considered keystone bacteria in the plant phyllosphere because their ability to fix carbon and nitrogen make them less dependent of the plant photosynthates, but also allow them to set the niche for the colonization of other heterotrophs (Rigonato et al., 2012; Thapa and Prasanna, 2018). Moreover, Cyanobacteria are pioneer microorganisms in desert soil crusts that contribute to carbon and nitrogen deposition (Powell et al., 2015). We propose that Cyanobacteria are key members of the phyllosphere of agaves and cacti by influencing the fitness of the heterotrophic microbial communities and contributing to the higher microbial diversity in native plants compared to cultivated *A. tequilana*.

Several of the photosystem I and II genes were assigned to Bacillales and Lactobacillales like *Staphylococcus* and *Trichococcus* respectively, which is remarkable because Heliobacteria represent the only known phototrophic clade of Firmicutes (Sattley et al., 2008). Since there is no evidence (strains or genomes) that these bacteria are indeed photosynthetic, we hypothesize that these genes might function for light sensing as suggested already by Finkel et al. (2016), whom conclude that light sensing in diverse lineages (such as Bacillales) is a relevant adaptation to colonize the phyllosphere of the dessert tree *Tamarix aphyla*.

The enrichment of reaction centers pufML, methanol dehydrogenase, carotene and bacteriochlorophyll biosynthesis indicates that AAP is a widespread trait in the phyllosphere of agaves and cacti. AAP bacteria can generate energy from light to survive starvation (Hanada, 2016), but they are not photoautotrophic and need to obtain carbon by fixing CO_2_ via anaplerotic pathways (Tang et al., 2009) or utilizing small carbon molecules like methanol (Müller et al., 2016). These bacteria are abundant in some oligotrophic environments like marine and freshwater (Hanada, 2016), but little attention has been paid to their presence in plant surfaces (Atamna-Ismaeel et al., 2012). *Methylobacterium* (Rhizobiales) scaffolds were the main contributors to this enrichment in native agave and cacti. This finding highlights the widespread presence and importance of *Methylobacterium* in the phyllosphere of most land plants. Moreover, members of this genus also exert positive effects on the health and growth of different plants (Hornschuh et al., 2002; Abanda-Nkpwatt et al., 2006; Hellmuth and Kutschera, 2008; Madhaiyan et al., 2009; Tani et al., 2012; Kwak et al., 2014; Madhaiyan and Poonguzhali, 2014; Krishnamoorthy et al., 2018). Contrary to native plants, in the phyllosphere of *A. tequilana* from Amatitan the main AAP bacteria were the genus *Belnapia* (Rhodospirillales) that has also been linked to AAP in desert microbial crusts (Csotonyi et al., 2010) and to growth promotion of *A. thaliana* (Camarena-Pozos et al., 2019). We propose that AAP *Methylobacterium* and *Belnapia* can be effective colonizers of the agave and cacti phyllosphere due to their ability to utilize both chemical and luminous energy, and also influence the growth and health of agaves and cacti.

### Metabolic Specialization in the Epiphytic Communities of Agaves and Cacti

Remarkably, rhizospheric prokaryotes differentiate from phyllospheric ones in the abundance of more diverse transporters (sugars, amino acids, opines, ions, etc.). These findings agree with the concept that the rhizosphere have richer and more diverse nutrient sources [e.g., root exudates (Vandenkoornhuyse et al., 2015)], while phyllospheric microorganisms of agaves and cacti might rely more on sugars and phototrophy to obtain energy. However, our analyses differ from a study on *A. thaliana* where, based on 400 bacterial genomes (Bai et al., 2015), authors inferred that the leaf microbiome rely on a more diverse and complex variety of carbon sources than the root microbiome.

Supplementary Figure S8 shows that *A. tequilana* is reduced in carbon metabolism genes compared to soil and other plant species (see also Supplementary File S1). The most notably lacking gene was the methanol dehydrogenase (mdh) that is a marker for methylotrophy. This process is relevant in the plant phyllosphere since plants emit high amounts of methanol from pectin demethylation (Thapa and Prasanna, 2018), but it was also a main signature in the phyllosphere of rice (Knief et al., 2012). The microbiome of *A. tequilana* might have less capabilities to rely on autotrophy and/or on the production, uptake and transport of diverse carbon sources since our analyses showed a reduction in genes related to methane, glyoxylate, propanoate and butanoate metabolism and carbon fixation compared to cultivated soils and one or more native plants (Supplementary Figure S7).

Besides carbon metabolism, the biosynthesis of secondary metabolites (vancomycin, ansamycins, PKS type II, NRPS, etc.) and xenobiotic degradation pathways (chloroalkane, chlorocyclohexane, aminobenzoate, steroid, xylene, etc.) were overrepresented in the phyllosphere and rhizosphere of native plants compared to *A. tequilana* (Figure 3 and Supplementary Figure S7). Depletion of these processes might be disadvantageous for the microbial community and the host fitness because some of these compounds might have antimicrobial activity against plant pathogens (Bulgarelli et al., 2013). Also, *A. tequilana* microbial communities might be less capable to cope with atmospheric and soil pollutants, since epiphytic bacteria can metabolize plant volatiles (isoprene, methanol) or other compounds that are toxic to plants and animals [e.g., polycyclic aromatic hydrocarbon, pesticides, etc. (Bringel and Couée, 2015)]. The causes and consequences of the loss of taxonomic and functional diversity and their influence on plant fitness in *A. tequilana* should be assessed by enriching, depleting or interchanging their epiphytic microbiomes using less complex synthetic communities as previously suggested (Busby et al., 2017; Vorholt et al., 2017).

### Nitrogen Fixation Is a Signature in the Rhizosphere of *A. tequilana*

Despite the functional losses in the *A. tequilana* microbiome, the nifH gene for nitrogen fixation was enriched in the rhizosphere of this plant species and it was linked to *Enterobacteriales* (Figure 4). Our findings agreed with the work of Knief et al. (2012), whom showed that the nitrogenase was found exclusively in the rhizospheric metaproteomes, although they found genes also in the phyllospheric metagenomes. Members of the *Enterobacteriales* have been isolated as nitrogen fixing bacteria from the rhizosphere and roots of *A. tequilana*, *A. salmiana* (Desgarennes et al., 2014) and also from the cultivated *A. sisalana* (Santos et al., 2014). These findings suggest that *A. tequilana* and other crops might be selecting beneficial microorganisms in their rhizospheres.

### Biofilm Formation in the Agaves and Cacti Epiphytic Microbiome

Biofilm formation pathways were enriched in both plant-associated communities, but not in the soil, suggesting that biofilm formation is a necessary trait for the interactions between the plant and its microbiome in arid and semiarid environments. Biofilms might allow microbial communities to aggregate and adhere to the plant tissues (Supplementary Figure S10B), increase water availability and the interchange of nutrients (e.g., fixed nitrogen in the rhizosphere of *A. tequilana*), genetic material and/or even virulence factors (Danhorn and Fuqua, 2007), as well as serving as protection against stress like desiccation and high UV radiation in arid environments.

Biosurfactant biosynthesis enriched genes such as srfAB have been implicated in the capability of *Bacillus* to trigger the formation of biofilms and its ability to suppress disease in the melon phyllosphere (Zeriouh et al., 2014), while rhamnolipids are involved in biofilm formation, surface mobility and the uptake of poorly soluble nutrients (Abdel-Mawgoud et al., 2010). Surfactants might represent an advantage to the phyllospheric communities since they increment the amount of photosynthates that are permeable in the cuticle-surface water interface (Schreiber, 2005).

Despite a significant functional reduction in the rhizosphere and phyllosphere of *A. tequilana*, biofilm formation was enriched and linked to diazotrophic lineages in this species (*Enterobacteriales*, Figures 4, 5). These findings suggest that biofilm is a required trait for the microbial communities to colonize, survive and exert a benefit to their host, and allow us to hypothesize how a less diverse microbiome can support the health and growth of *A. tequilana.*

In sum, this work sheds light into the potential mechanisms by which above- and below-ground epiphytic microbial communities survive and colonize plants of arid and semiarid ecosystems. Congruent with our previous works, we found functional differentiation in the microbial communities of the soil, rhizosphere and phyllosphere of agaves and cacti. Despite the low replication level in this metagenomic study, we identified the likely loss of several microbial functions in the episphere of cultivated *A. tequilana*. All these findings will serve as baseline for the design of microbial synthetic communities. These communities will allow us to test if the enrichment and/or depletion of specific functional groups (biofilm-producing, phototrophic bacteria, diazotrophs) influence microbial diversity and/or the growth, health and productivity of cultivated agaves in the field.

## Data Availability Statement

The datasets generated for this study can be found in the IMG platform, IMG Genome IDs 3300032159, 3300030499, 3300030692, 3300030502, 3300030753, 3300030500, 3300030504, 3300030512, 3300030505, 3300030514, 3300030501, 3300030497, 3300030498, 3300030516, 3300030495, 3300030515, 3300030511, 3300030510, 3300030496, and 3300030513.

## Author Contributions

VF-N and LP-M planned and designed the research, analyzed the data, and wrote the manuscript. CF-G, DD, and LP-M selected the sampling sites, and collected and prepared the metagenomic samples of *M. geometrizans*, *O. robusta, A. salmiana, A. tequilana*, and bulk soils. EE-F and TW prepared the libraries, and processed and annotated the sequencing data. VF-N performed the metagenomic analyses, isolated and characterized the sequenced bacterial strains, and created the figures. LP-M secured the funding. All authors read and approved the final manuscript.

## Conflict of Interest

The authors declare that the research was conducted in the absence of any commercial or financial relationships that could be construed as a potential conflict of interest.
