# Supplementary material for: Functional Signatures of the Epiphytic Prokaryotic Microbiome of Agaves and Cacti
Source: Front Microbiol. 2020 Jan 17;10:3044. doi: 10.3389/fmicb.2019.03044 (PMC6978686; doi:10.3389/fmicb.2019.03044)
Supplement: Supplementary file 1 [file Presentation_1.pdf]

Supplementary Material

Functional signatures of the epiphytic prokaryotic microbiome of agaves and cacti

Víctor M. Flores-Núñez, Citlali Fonseca-García, Damaris Desgarennes, Emiley Eloe-Fadrosh, Tanja Woyke and Laila P. Partida-Martínez*

*** Correspondence:** Laila P. Partida-Martinez (laila.partida@cinvestav.mx)

Supplementary Methods

Isolation and characterization of strains

Leaves of *A. salmiana* and *A. tequilana* were collected from El Magueyal and Penjamo, Guanajuato, respectively, in September of 2017. The phyllosphere was recovered following the protocol of Desgarennes et al., (2014). For the enrichment of methylotrophic bacteria, the phyllosphere suspensions were ten-fold diluted and inoculated in liquid Czapek-Dox media (2g $NaNO_3$, 1g K_2HPO_4, 0.5g $MgSO_4$, 0.5g KCl, 0.01g $FeSO_4$, per liter) with methanol 123mM (final concentration) and subcultured every week during 3 weeks until pink colored cultures developed. These cultures were ten-fold diluted and plated in tryptic soy agar (TSA). Pink colored colonies of Gram-negative bacteria were selected. The diluted phyllosphere suspensions were also inoculated in Gromov No. 6 media (Temraleeva et al., 2016). Flasks were incubated in a room with photoperiod (16h light/8h darkness) at 28°C and low speed agitation. After two months of incubation, only *A. tequilana* samples showed cyanobacterial growth. In order to obtain pure and axenic cultures, subcultures of cyanobacterial filaments were made in the same media every week during 9 weeks. Microscopic morphology was assessed in 2 week-old cultures.

For both pink methylotrophic bacteria and cyanobacteria, the 16S rRNA genes were amplified and cloned using the pGEM-T easy cloning kit (PROMEGA) in *E. coli* JM109 and sequenced as described in (Desgarennes et al., 2014). 16S rRNA homologous sequences were retrieved from BLASTn, then aligned using MUSCLE (Edgar, 2004), trimmed and the sequence percentage of identity was calculated based on the variable bases (Table S5).

Genome sequencing

High quality genomic DNA *Methylobacterium* RAS18, *Belnapia rosea* MJ22 and *Kosakonia sacchari* MJ18 was extracted from 1-3 day-old monoclonal cultures in TSB as described in Desgarennes et al., (2014). Genome sequencing was performed at the Joint Genome Institute (JGI) using an Illumina HiSeq 2500-1TB instrument. Raw reads were processed using the custom pipeline developed by the JGI. Quality reads were assembled using ALLPATHS v. r46652 (MJ18 and MJ22) (Ribeiro et al., 2012) and Spades 3.12.0 (RAS18) (Bankevich et al., 2012), the resulting scaffolds were annotated using the IMG Annotation Pipeline v.4.16.5 (Huntemann et al., 2016).

Supplementary Files

File S1 (Table 1.xlsx). Gene enrichment analysis summary. Each spreadsheet contains a list of genes in the KEGG pathway database (pathway, enzyme, ko, name) that are enriched (colored cells, FDR <0.05) in the comparisons between plant compartments (grey) and species (brown). Cells with “0” indicate not enriched genes.

Supplementary Figures

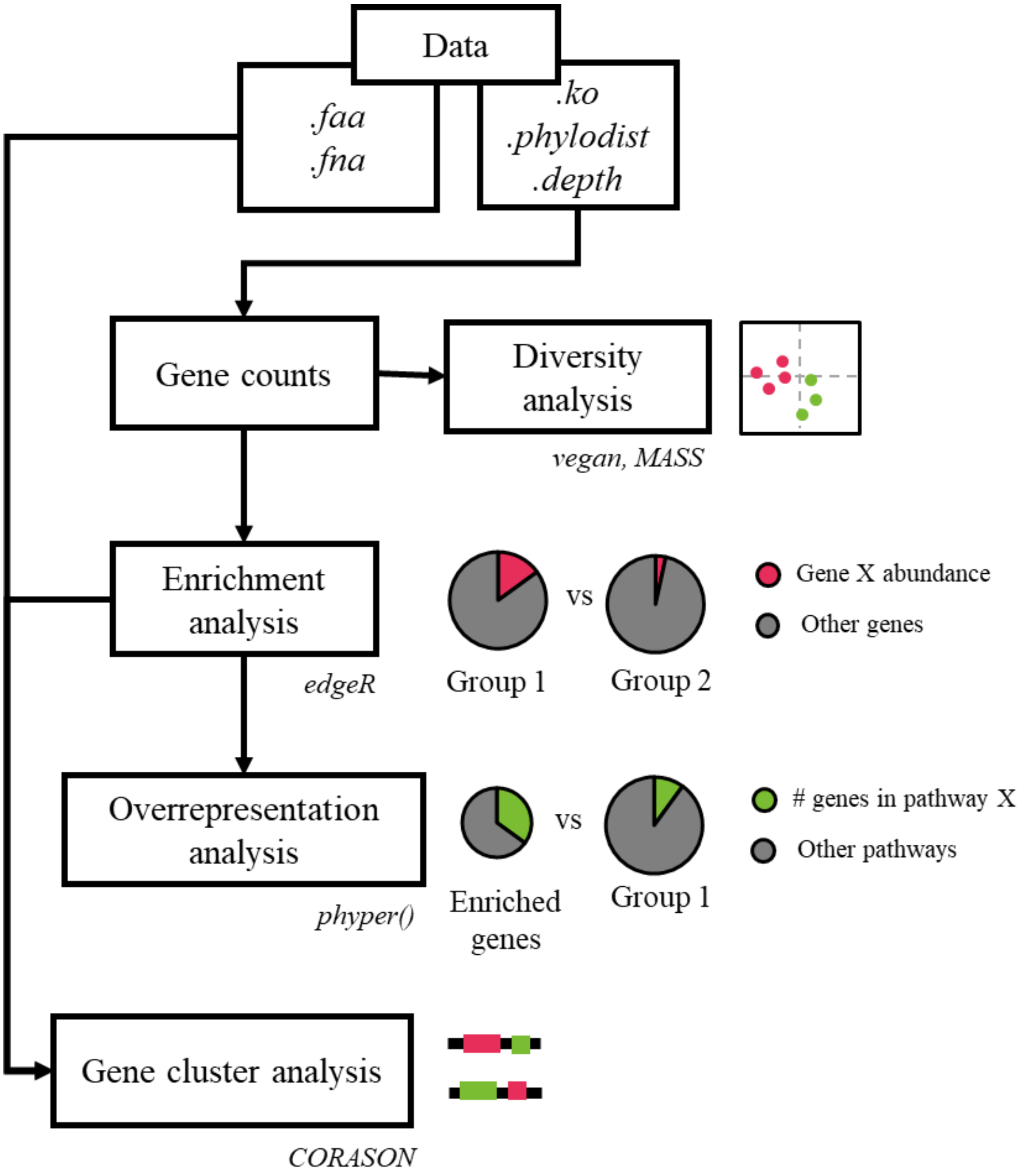

Figure S1. Statistical analysis overview. Datasets are available in the IMG platform. Code for gene counting, diversity, enrichment and overrepresentation analysis are available at github.com/vicflonun/Agaviromics/. CORASON is available at github.com/nselem/corason/

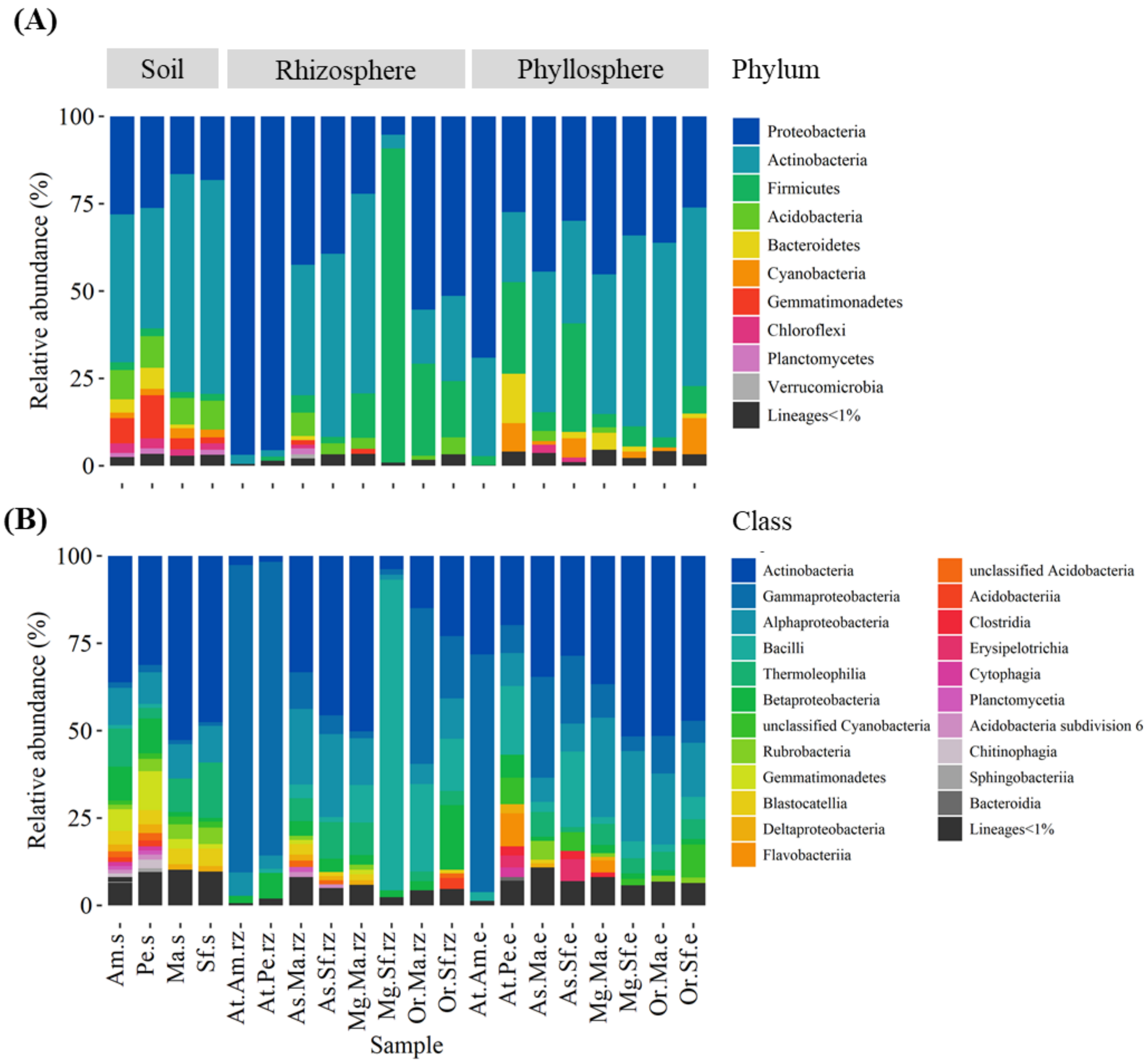

Figure S2. Taxonomic annotation of bacterial genes across samples. Estimated gene copies of **(A)** each bacterial phylum and **(B)** class were normalized to the number of total copies in each dataset. Unassigned genes were not considered. Species: At - *A. tequilana*, As - *A. salmiana*, Mg - *M. geometrizans*, Or - *O. robusta*. Sites: Am - Amatitan, Pe - Penjamo, Ma - El Magueyal, Sf - San Felipe. Compartment: s - bulk soil, rz - rhizosphere, e - phyllosphere. Lineages <1% - low abundant taxa.

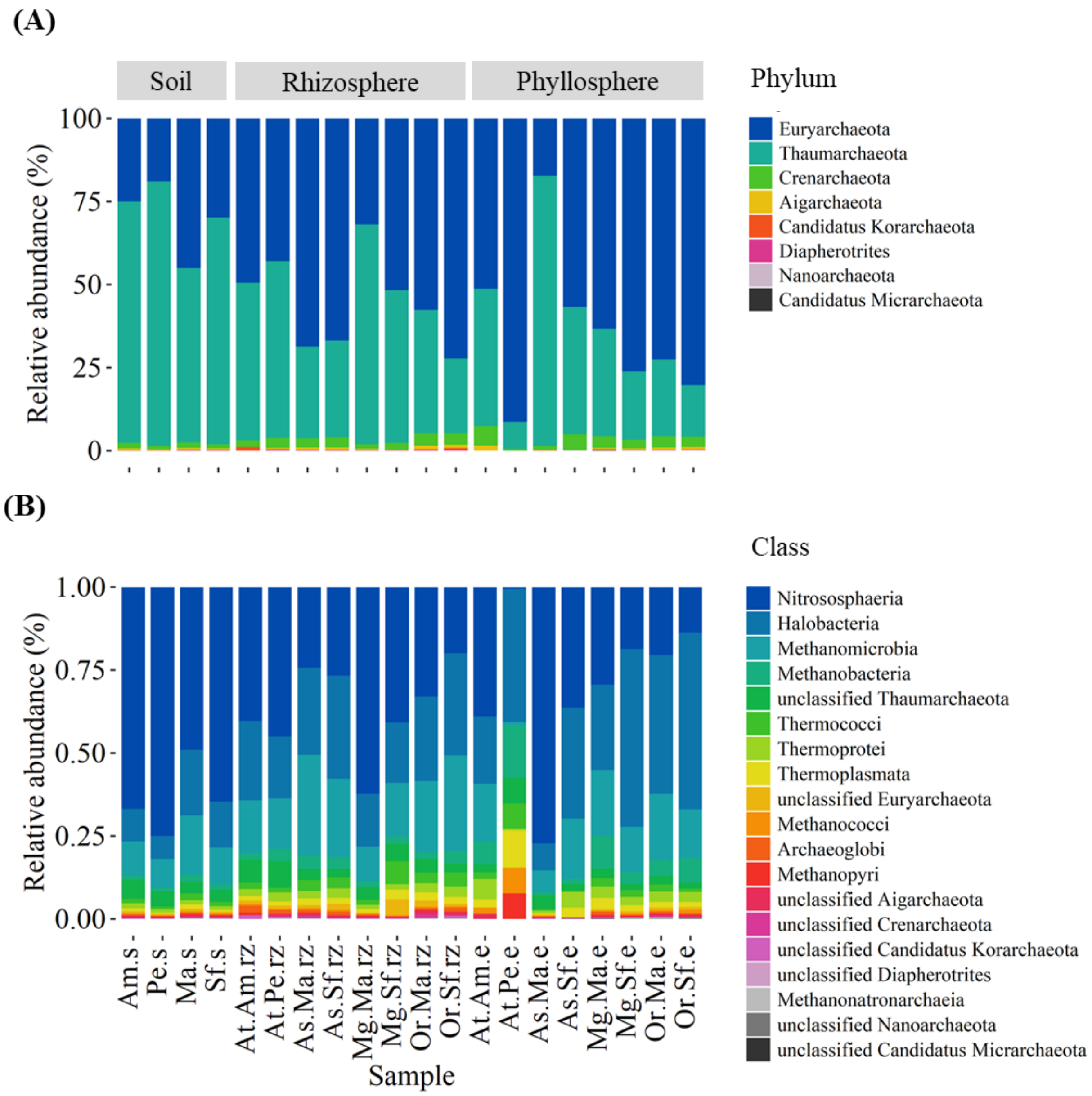

Figure S3. Taxonomic annotation of archaeal genes across samples. Estimated gene copies of **(A)** each archaeal phylum and **(B)** class were normalized to the number of total copies in each dataset. Unassigned genes were not considered. Species: At - *A. tequilana*, As - *A. salmiana*, Mg - *M. geometrizans*, Or - *O. robusta*. Sites: Am - Amatitan, Pe - Penjamo, Ma - El Magueyal, Sf - San Felipe. Compartment: s - bulk soil, rz - rhizosphere, e - phyllosphere.

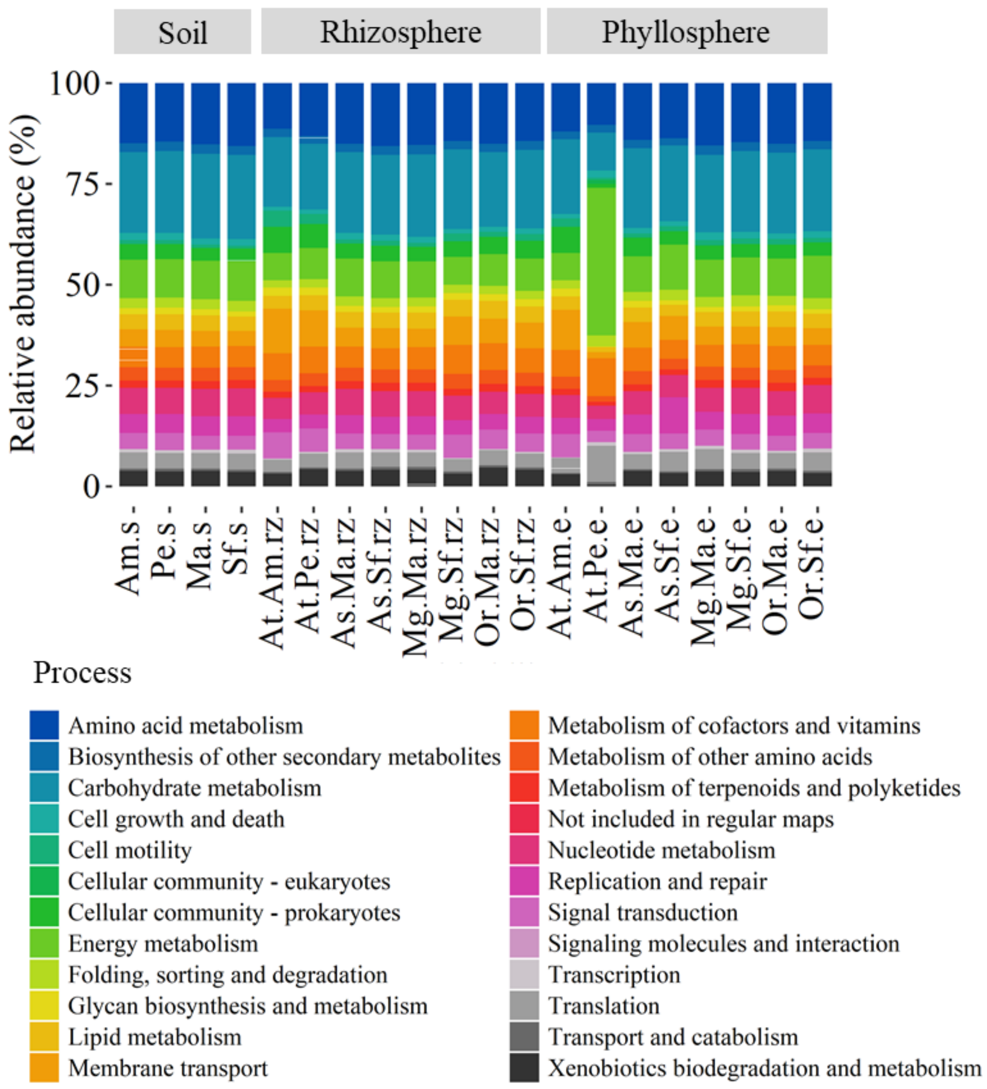

Figure S4. Functional annotation by process of prokaryotic genes across samples. Estimated gene copies of each metabolic pathway in KEGG database was normalized to the number of total copies in each dataset. Species: At - *A. tequilana*, As - *A. salmiana*, Mg - *M. geometrizans*, Or - *O. robusta*. Sites: Am - Amatitan, Pe - Penjamo, Ma - El Magueyal, Sf - San Felipe. Compartment: s - bulk soil, rz - rhizosphere, e - phyllosphere.

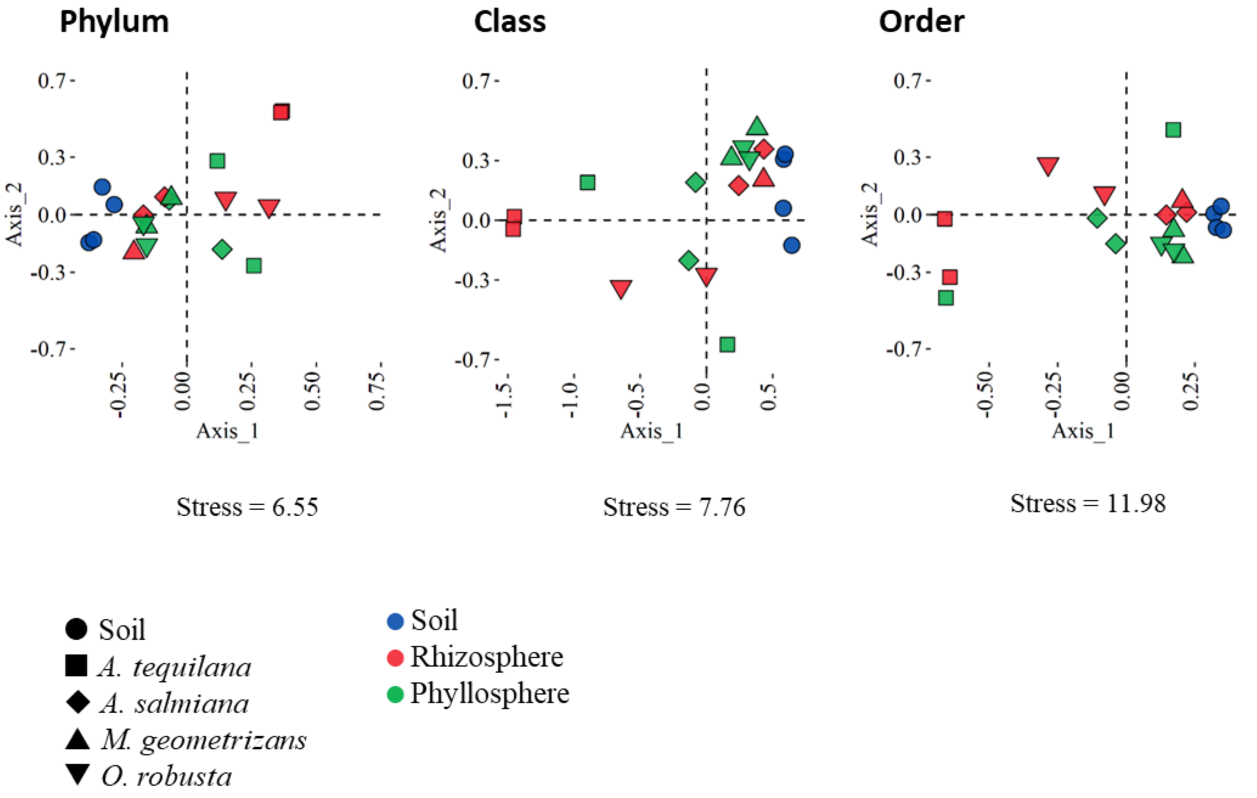

Figure S5. Nonmetric multidimensional scaling ordination of Bray-Curtis dissimilarities between samples. The proximity between points represents the similarity between samples based on the rarified gene counts at the phylum, class and order level.

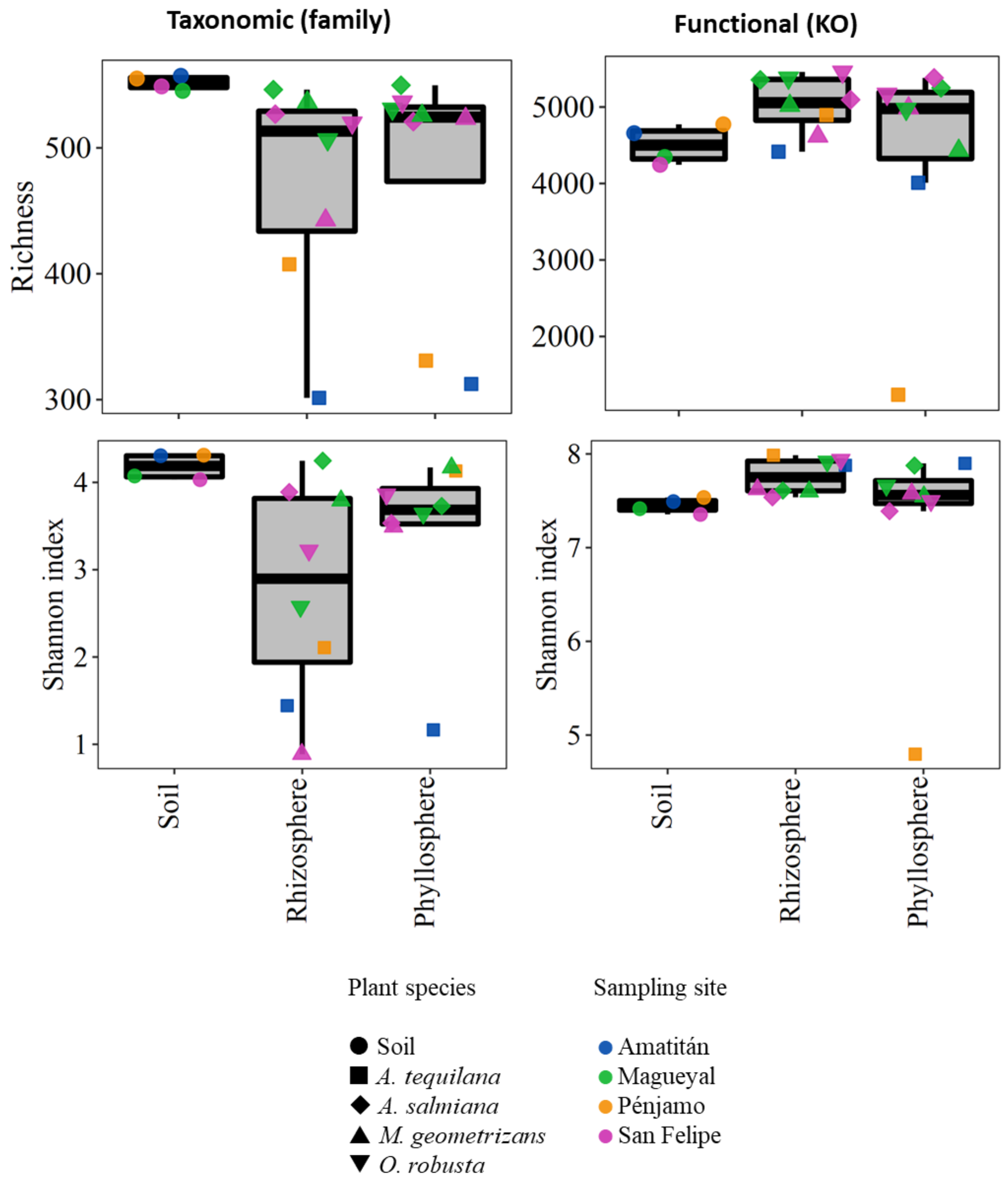

Figure S6. Alpha diversity indexes across samples. Taxonomic and functional diversity were calculated using the rarified gene counts (family level and KO, respectively)

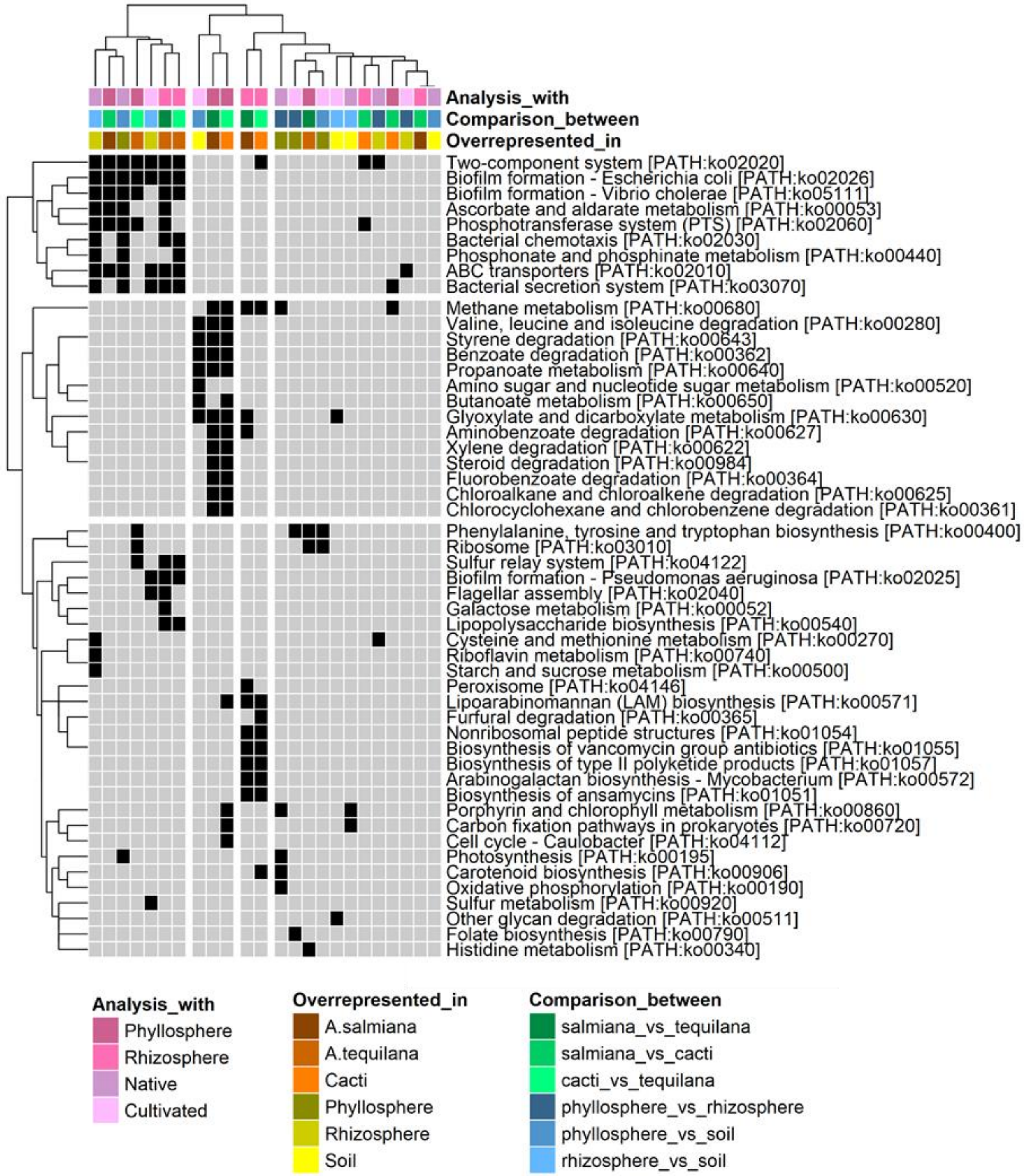

Figure S7. Pathway overrepresentation of the differential enriched genes. Each black square represents an overrepresented pathway (rows) found in each pairwise comparison between compartments and plants (columns).

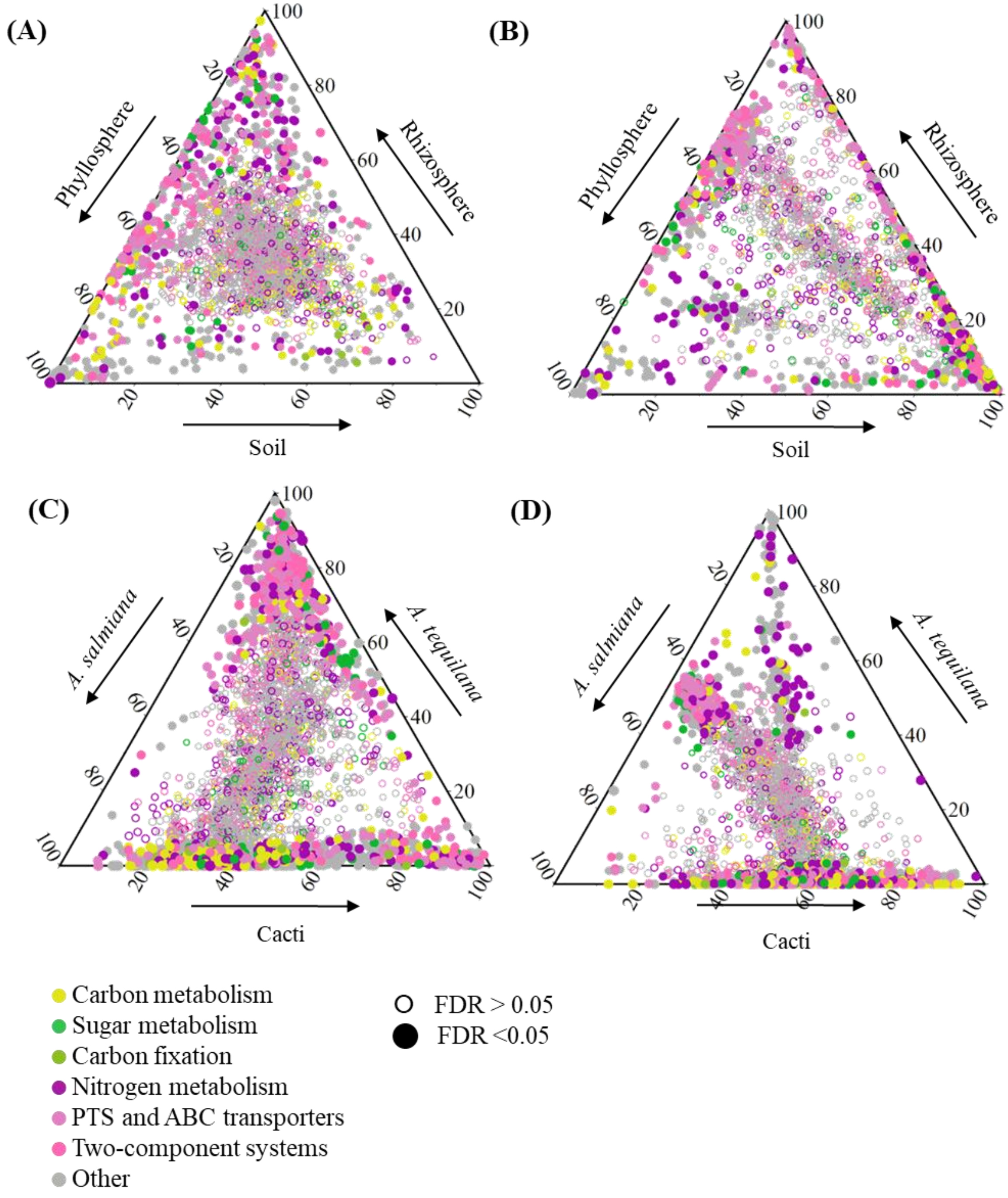

Figure S8. Ternary plot of differential enriched genes. Dots represent genes which position is defined by the ratio of its mean abundance between plant compartments (top panel) or species (bottom panel). Genes were colored based on functional groups of KO annotation and their shape is based on their FDR value in each pairwise comparison. Comparisons were made between compartments for **(A)** native sympatric plants and **(B)** *A. tequilana*; and between plant species for

(C) the rhizosphere and **(D)** phyllosphere. Arrows represent the direction of the enrichment. Carbon metabolism: methane metabolism [ko00680], glyoxylate and dicarboxylate metabolism [ko00630], propanoate metabolism [ko00640], butanoate metabolism [ko00650]. Sugar metabolism: fructose and mannose metabolism [ko00051], starch and sucrose metabolism [ko00500]. Carbon fixation: carbon fixation pathways in prokaryotes [ko00720], carbon fixation in photosynthetic organisms [ko00710]. Nitrogen metabolism: nitrogen metabolism [ko00910] and amino acid metabolism pathways (ko00250-00400). PTS and ABC transporters: ABC transporters [ko02010], phosphotransferase system (PTS) [ko02060]. Two-component system: two-component system [ko02020].

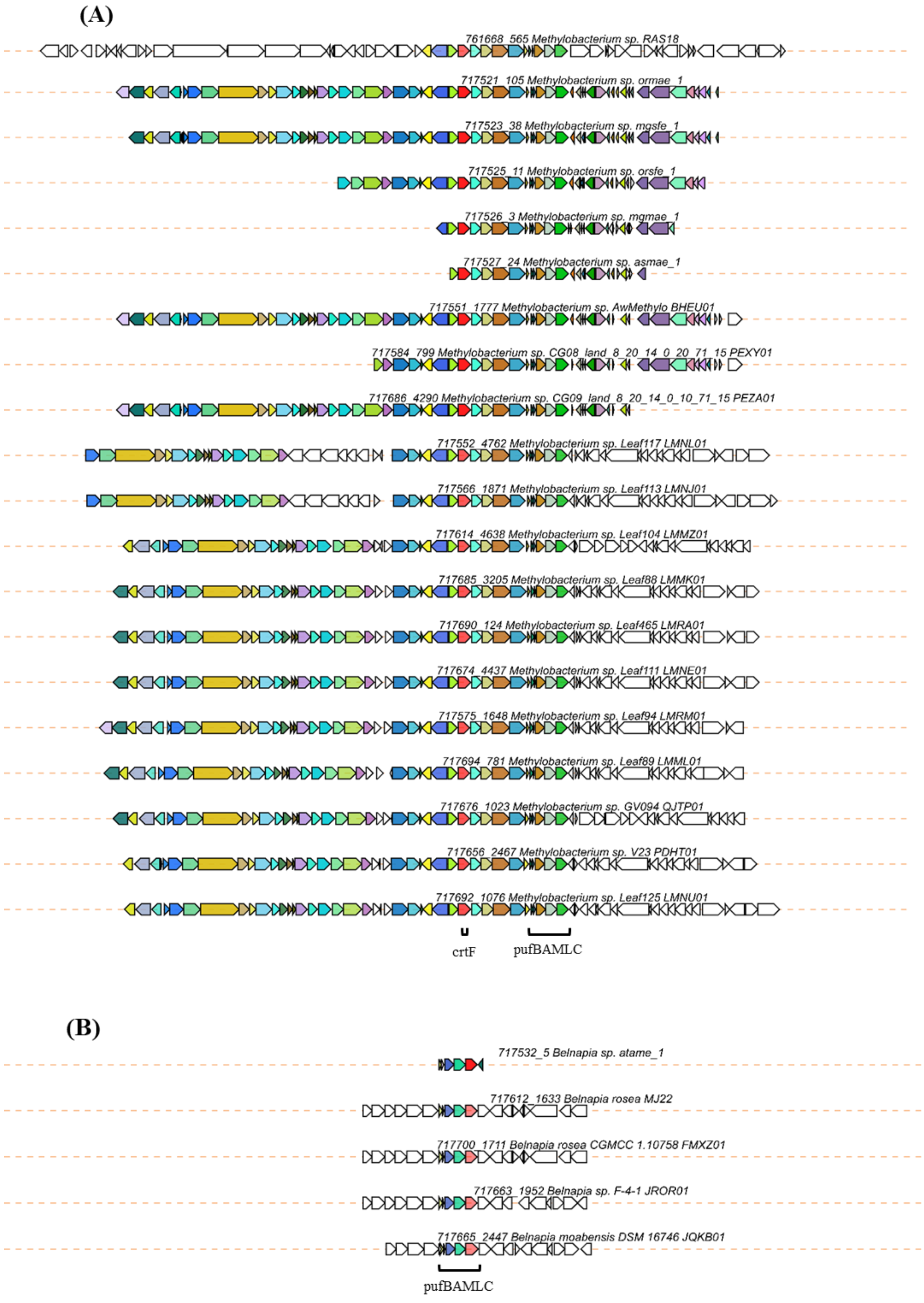
(A)
761668_565 Methylobacterium sp. RAS18
717521_105 Methylobacterium sp. ormae_1
717523_38 Methylobacterium sp. mgsfe_1
717525_11 Methylobacterium sp. orsfe_1
717526_3 Methylobacterium sp. mgmae_1
717527_24 Methylobacterium sp. asmae_1
717551_1777 Methylobacterium sp. AwMethylo BHEU01
717584_799 Methylobacterium sp. CG08_land_8_20_14_0_20_71_15 PEXY01
717686_4290 Methylobacterium sp. CG09_land_8_20_14_0_10_71_15 PEZA01
717552_4762 Methylobacterium sp. Leaf117 LMNL01
717566_1871 Methylobacterium sp. Leaf113 LMNJ01
717614_4638 Methylobacterium sp. Leaf104 LMMZ01
717685_3205 Methylobacterium sp. Leaf88 LMMK01
717690_124 Methylobacterium sp. Leaf465 LMRA01
717674_4437 Methylobacterium sp. Leaf111 LMNE01
717575_1648 Methylobacterium sp. Leaf94 LMRM01
717694_781 Methylobacterium sp. Leaf89 LMML01
717676_1023 Methylobacterium sp. GV094 QJTP01
717656_2467 Methylobacterium sp. V23 PDHT01
717692_1076 Methylobacterium sp. Leaf125 LMNU01
crtF
pufBAMLC
(B)
717532_5 Belnapia sp. atame_1
717612_1633 Belnapia rosea MJ22
717700_1711 Belnapia rosea CGMCC 1.10758 FMXZ01
717663_1952 Belnapia sp. F-4-1 JROR01
717665_2447 Belnapia moabensis DSM 16746 JQKB01
pufBAMLC

Figure S9. Analysis of AAP gene clusters. **(A)** Cluster comparison based on the crtF gene in *Methylobacterium* scaffolds and *Methylobacterium* spp. genomes. **(B)** Cluster comparison based on the pufC gene in *Belnapia* scaffolds and *Belnapia* spp. genomes. Orthologous genes are indicated in colors.

(A)

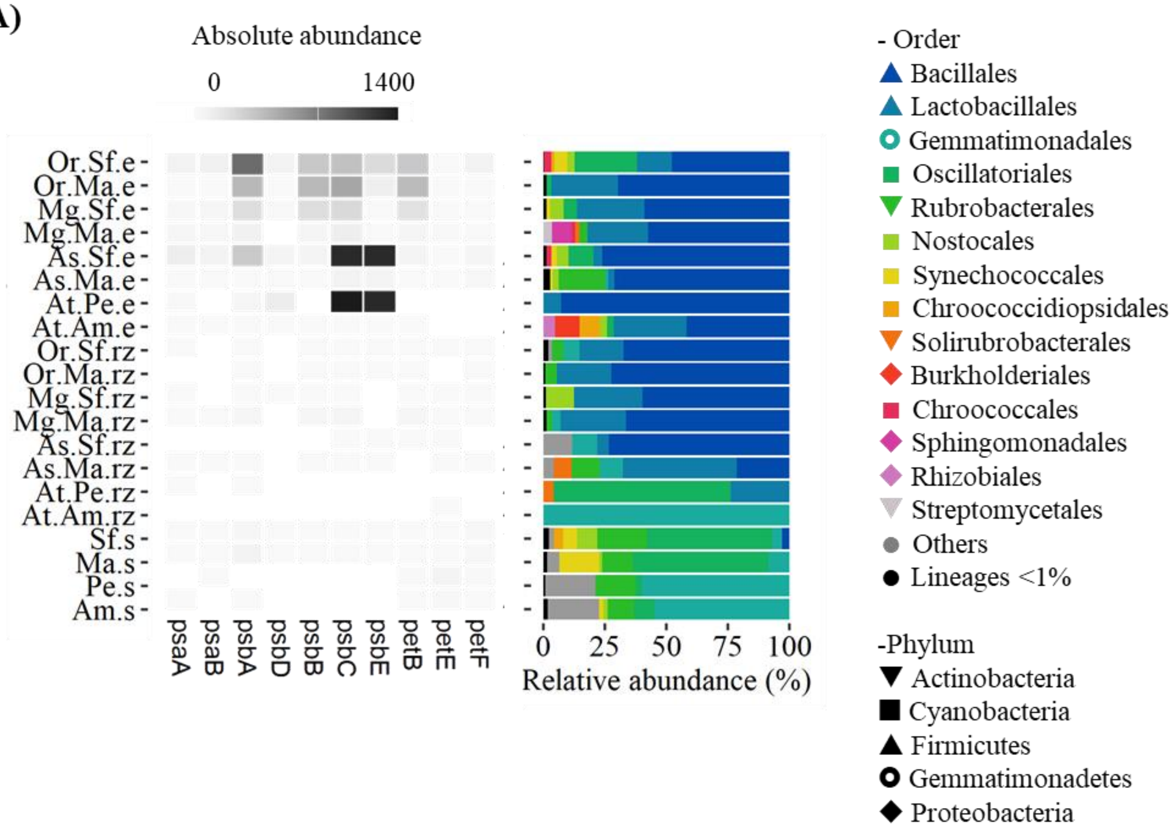

(B)

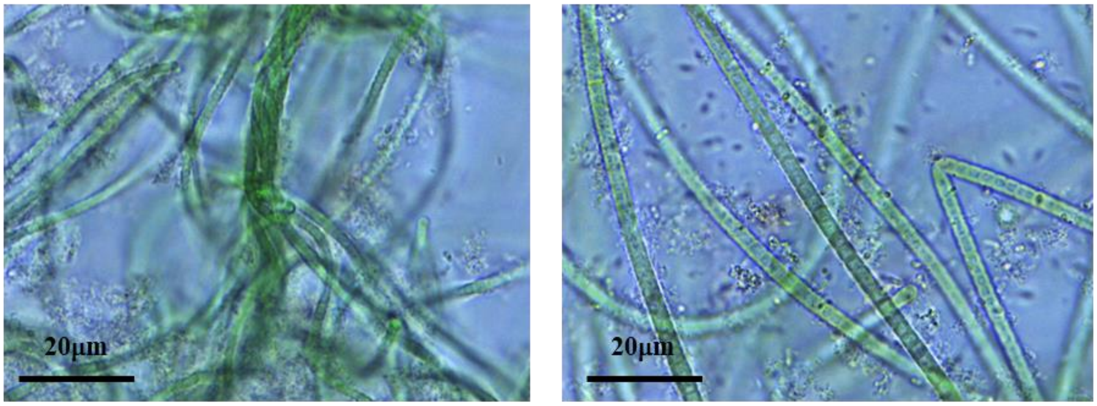

Nodosilinea sp. AT21. 100X

Leptolyngbya sp. AT32. 100X

Figure S10. Oxygenic photosynthesis profiling. **(A)** Abundance and taxonomic profile of the differential enriched photosystems (psa and psb) and cytochrome (pet) genes. **(B)** Bright field microscopy of the cyanobacterial isolates from the phyllosphere of *A. tequilana*. Species: At - *A. tequilana,* As - *A. salmiana,* Mg - *M. geometrizans*, Or - *O. robusta*. Sites: Am - Amatitan, Pe - Penjamo, Ma - El Magueyal, Sf - San Felipe. Compartment: s - bulk soil, rz - rhizosphere, e - phyllosphere. Others - taxa not present in the phyllosphere. Lineages <1% - low abundant taxa.

(A)

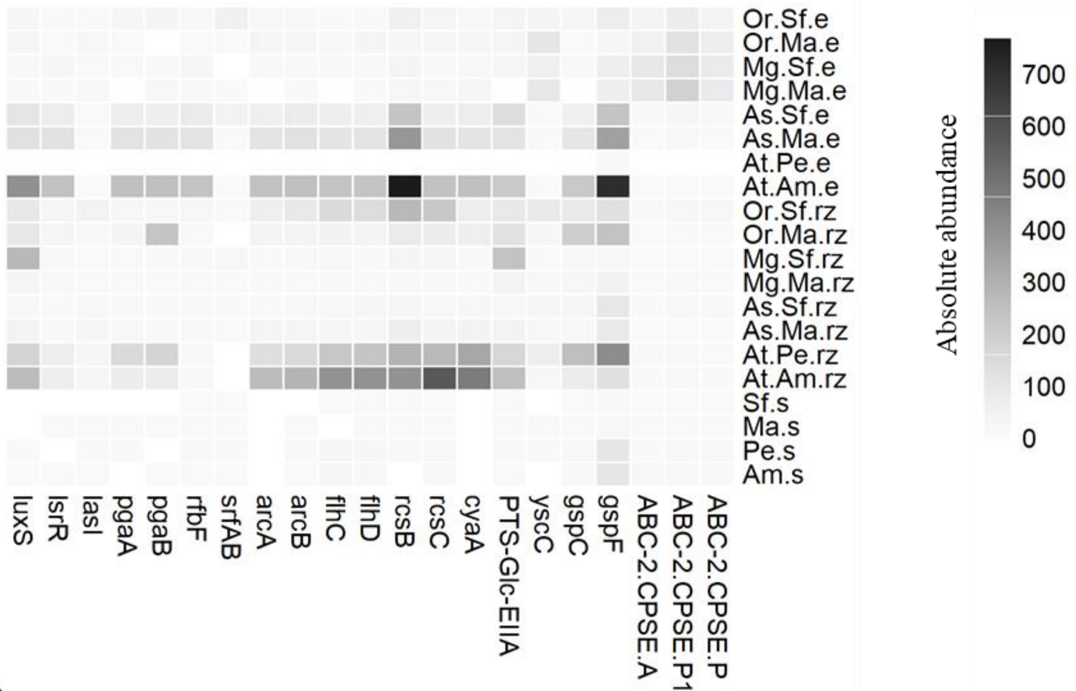

(B)

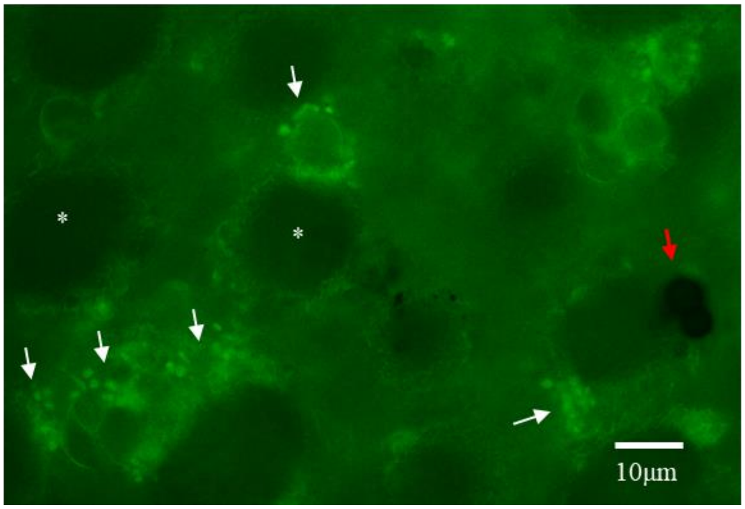

Figure S11. Biofilm and quorum sensing profiling. **(A)** Abundance profile of the differential enriched genes: quorum sensing (lasI, lsrR, luxS), matrix (pga, srf, rfb), regulators (arc, cya, flh, rcs, PTS-Glu), excretion systems (gsp, ycs) and ABC capsule transporters. **(B)** Fluorescence microscopy of the phyllosphere of *A. tequilana* stained with acridine orange 0.01%, 100x. White arrows indicate the presence of prokaryotic aggregates, the red arrow points a fungal spore and the white asterisks indicate leaf epidermal cells. Species: At - *A. tequilana,* As - *A. salmiana,* Mg - *M. geometrizans*, Or - *O. robusta*. Sites: Am - Amatitan, Pe - Penjamo, Ma - El Magueyal, Sf - San Felipe. Compartment: s - bulk soil, rz - rhizosphere, e - phyllosphere.

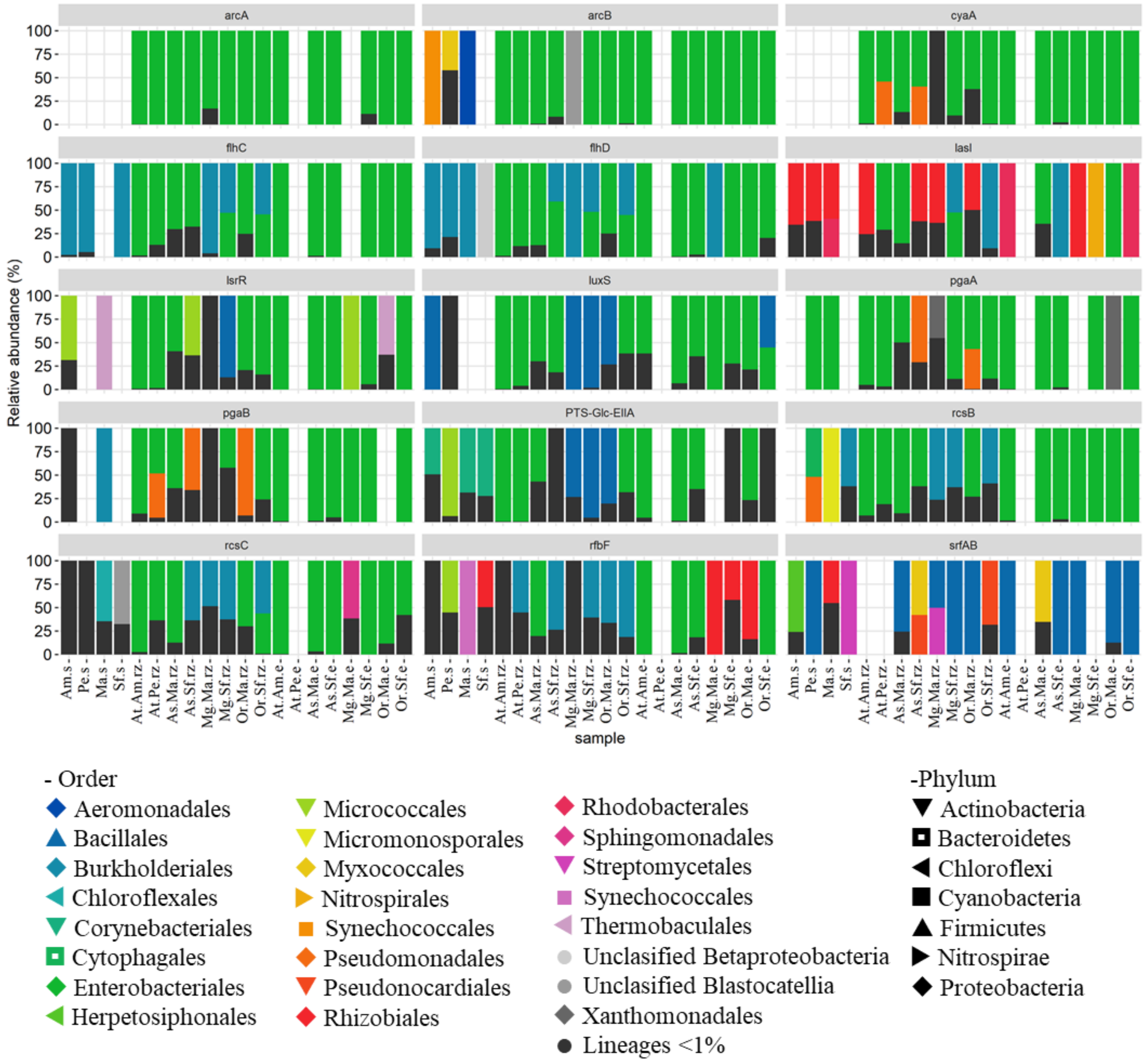

Figure S12. Taxonomic profile of the differential enriched genes of biofilm formation and quorum sensing. Quorum sensing (lasI, lsrR, luxS), matrix (pga, srf, rfb) and regulators (arc, cya, flh, rcs, PTS-Glu). Species: At - *A. tequilana,* As - *A. salmiana,* Mg - *M. geometrizans*, Or - *O. robusta*. Sites: Am - Amatitan, Pe - Penjamo, Ma - El Magueyal, Sf - San Felipe. Compartment: s - bulk soil, rz - rhizosphere, e - phyllosphere. Lineages <1% - low abundant taxa.

Supplementary Tables

Table S1. Library information.

Library ID	IMG Genome ID	NCBI Bioproject	NCBI Biosample Accession	SRA	Release Date	Library Size (bp)	Gene Count	Scaffold Count	Coding Base Count	CDS Count
**Am.s**	3300030511	PRJNA465696	SAMN12740640	SRP221822	15/12/2018	489,358,821	1,343,249	1,140,791	487,521,208	1,333,442
**Pe.s**	3300030496	PRJNA465697	SAMN12740641	SRP221820	15/12/2018	489,012,473	1,350,744	1,170,074	487,393,055	1,342,011
**Ma.s**	3300030510	PRJNA404245	SAMN12740642	SRP221823	15/12/2018	417,105,684	1,080,987	905,022	415,830,697	1,074,717
**Sf.s**	3300030513	PRJNA404246	SAMN12740643	SRP221824	17/12/2018	381,418,018	1,028,866	887,161	379,943,450	1,021,950
**As.Ma.e**	3300032159	PRJNA340601	SAMN05720245	SRP085235	23/01/2019	1,541,794,765	3,621,863	3,626,996	1,539,581,945	3,609,054
**As.Ma.rz**	3300030499	PRJNA340593	SAMN05720246	SRP085215	15/12/2018	495,573,182	1,313,267	1,116,488	493,986,918	1,305,152
**As.Sf.e**	3300030692	PRJNA340602	SAMN05720286	SRP085220	19/12/2018	1,770,074,211	3,289,843	3,798,103	1,768,470,884	3,281,095
**As.Sf.rz**	3300030502	PRJNA340594	SAMN05720244	SRP085216	15/12/2018	495,529,288	1,350,514	1,162,637	494,008,811	1,343,369
**At.Am.e**	3300030753	PRJNA340604	SAMN05720239	SRP221821	24/12/2018	66,113,247	123,147	80,406	65,863,503	121,756
**At.Am.rz**	3300030500	PRJNA340596	SAMN05720242	SRP085218	15/12/2018	291,451,256	495,277	288,868	290,881,342	490,959
**At.P.e**	3300030504	PRJNA340603	SAMN05720143	SRP085238	15/12/2018	173,440,829	267,476	326,958	173,203,562	266,333
**At.P.rz**	3300030512	PRJNA340595	SAMN05720243	SRP085217	17/12/2018	451,196,603	816,650	513,116	450,172,563	809,394
**Mg.Ma.e**	3300030505	PRJNA340607	SAMN05720169	SRP085255	15/12/2018	653,701,676	1,186,283	1,440,946	653,273,407	1,183,677
**Mg.Ma.rz**	3300030514	PRJNA340599	SAMN05720235	SRP085219	17/12/2018	830,522,015	2,017,114	1,834,725	828,593,871	2,006,500
**Mg.Sf.e**	3300030501	PRJNA340608	SAMN05720237	SRP221825	15/12/2018	1,667,191,862	3,467,797	3,414,274	1,665,560,895	3,456,959
**Mg.Sf.rz**	3300030497	PRJNA340600	SAMN05720240	SRP085223	15/12/2018	426,598,694	873,484	639,026	425,604,668	866,671
**Or.Ma.e**	3300030498	PRJNA340605	SAMN05720238	SRP096465	15/12/2018	1,080,334,363	1,942,137	2,088,047	1,079,364,888	1,935,725
**Or.Ma.rz**	3300030516	PRJNA340597	SAMN05720241	SRP085222	17/12/2018	594,623,726	1,265,171	999,417	593,214,311	1,256,419
**Or.Sf.e**	3300030495	PRJNA340606	SAMN05720236	SRP085254	15/12/2018	631,553,214	1,246,953	1,222,792	630,684,157	1,241,806
**Or.Sf.rz**	3300030515	PRJNA340598	SAMN05720285	SRP085221	17/12/2018	681,601,678	1,589,345	1,255,698	680,005,215	1,579,259

Nomenclature: **At -** *Agave tequilana*, **As** – *Agave salmiana*, **Mg** – *Myrtillocactus geometrizans*, **Or** – *Opuntia robusta*, **Am** – Site Amatitan, **P** or **Pe** – Site Penjamo, **Ma** – Site Magueyal, **Sf** – Site San Felipe, **s** – soil, **rz** – rhizosphere and **e** – phyllosphere.

Table S2. Gene enrichment analysis overview. Colors depend on the sample group considered.

Comparison		Sample groups	No. of samples	No. enriched genes in all possible comparisons	
Between compartments	Native	Phyllosphere	6	158	357
		Rhizosphere	6	209	399
		Soil	2	34	57
	A. tequilana	Phyllosphere	2	80	255
		Rhizosphere	2	75	237
		Soil	2	247	184
Between species	Rhizosphere	*A. tequilana*	2	447	399
		A. salmiana	2	646	8
		Cacti	4	769	110
	Phyllosphere	*A. tequilana*	2	108	565
		A. salmiana	2	463	211
		Cacti	4	657	27
Between cacti	Rhizosphere	*O. robusta*	2	1	
		M. geometrizans	2	0	
	Phyllosphere	*O. robusta*	2	21	
		M. geometrizans	2	8	
Between soil		Cultivated soil	2	31	
		Native soil	2	16	

Table S3. Genomes retrieved from NCBI database for CORASON analysis.

Name	Assembly	Name	Assembly
**Enterobacter sp. R4-368**	GCA_000410515.1	**Methylobacterium sp. Leaf465**	GCA_001424705.1
**Enterobacter sp. EA-1**	GCA_002886105.1	**Methylobacterium brachiatum 111MFTsu3.1M4**	GCA_900113845.1
**Enterobacter sp. Bisph2**	GCA_000814915.1	**Methylobacterium sp. Leaf125**	GCA_001423085.1
**Enterobacter sp. 10-1**	GCA_002270295.1	**Methylobacterium sp. Leaf91**	GCA_001422815.1
**Enterobacter sp. 9-2**	GCA_003353115.1	**Methylobacterium sp. Leaf113**	GCA_001423325.1
**Enterobacter sp. AG5470**	GCA_004379835.1	**Methylobacterium sp. Leaf88**	GCA_001422795.1
**Enterobacter sp. NFIX09**	GCA_900111655.1	**Methylobacterium sp. AP11**	GCA_900110515.1
**Enterobacter sp. NFIX04**	GCA_900112805.1	**Methylobacterium sp. Leaf90**	GCA_001422265.1
**Enterobacter sp. NFIX08**	GCA_900115965.1	**Methylobacterium sp. GV094**	GCA_003217615.1
**Enterobacter sp. NFIX06**	GCA_900116465.1	**Methylobacterium sp. Leaf87**	GCA_001422775.1
**Enterobacter sp. NFIX03**	GCA_900119515.1	**Methylobacterium sp. B4**	GCA_003201865.1
**Enterobacter sp. NFR05**	GCA_900168315.1	**Methylobacterium sp. Leaf100**	GCA_001422845.1
**Enterobacter sp. FY-07**	GCA_001582075.1	**Methylobacterium sp. GV104**	GCA_003053915.1
**Enterobacter cloacae CZ-1**	GCA_004193715.1	**Methylobacterium sp. Leaf94**	GCA_001426045.1
**Enterobacter cloacae TUM10**	GCA_003175235.1	**Methylobacterium sp. NP6**	GCA_002727035.1
**Enterobacter cloacae TUM1144**	GCA_003175375.1	**Methylobacterium sp. 275MFSha3.1**	GCA_900108435.1
**Enterobacter cloacae TUM1506**	GCA_003175745.1	**Methylobacterium sp. 13MFTsu3.1M2**	GCA_900112625.1
**Kosakonia oryzae Ola 51**	GCA_001658025.1	**Methylobacterium sp. Leaf111**	GCA_001423285.1
**Kosakonia oryzendophytica LMG 26432T**	GCA_900185945.1	**Methylobacterium sp. UNC378MF**	GCA_900103195.1
**Kosakonia oryziphila REICA_142**	GCA_900094795.1	**Methylobacterium gossipiicola Gh-105**	GCA_900113485.1
**Kosakonia arachidis Ah-143**	GCA_900116535.1	**Methylobacterium sp. UNC300MFChir4.1**	GCA_900110155.1
**Kosakonia pseudosacchari NN143**	GCA_002510255.1	**Methylobacterium phyllosphaerae CBMB27**	GCA_900113465.1
**Kosakonia pseudosacchari JM-387 T**	GCA_900184035.1	**Methylobacterium sp. UNCCL125**	GCA_900116795.1
**Kosakonia sp. H7A**	GCA_003028445.1	**Methylobacterium sp. 190MF**	GCA_900107945.1
**Kosakonia oryzendophytica REICA_082**	GCA_900094925.1	**Methylobacterium sp. YR596**	GCA_900112945.1
**Kosakonia oryzae D4**	GCA_900168185.1	**Methylobacterium organophilum DSM 760**	GCA_003096615.1
**Kosakonia radicincitans UMEnt01/12**	GCA_000691205.1	**Methylobacterium sp. 174MFSha1.1**	GCA_900116815.1
**Kosakonia oryzae KO348**	GCA_000958895.1	**Methylobacterium sp. ARG-1**	GCA_001262595.1
**Kosakonia sp. AG348**	GCA_003337365.1	**Methylobacterium sp. 285MFTsu5.1**	GCA_000383455.1
**Kosakonia oryzae CGMCC 1.7012**	GCA_900112145.1	**Methylobacterium sp. Leaf361**	GCA_001424445.1

**Kosakonia sp. S29**	GCA_900112785.1	**Methylobacterium sp. YR668**	GCA_900116245.1
**Kosakonia radicincitans DSM 16656**	GCA_000280495.2	**Methylobacterium sp. 6HR-1**	GCA_004745635.1
**Kosakonia sacchari SP1**	GCA_000300455.4	**Methylobacterium sp. AwMethylo**	GCA_003864615.1
**Kosakonia sacchari BO-1**	GCA_001683395.1	**Methylobacterium sp. GXS13**	GCA_001455965.1
**Kosakonia radicincitans GXGL-4A**	GCA_001887675.1	**Methylobacterium sp. CG09_land_8_20_14_0_10_71_15**	GCA_002778835.1
**Kosakonia sacchari HX148**	GCA_002510275.1	**Methylobacterium sp. GXF4**	GCA_000272495.1
**Kosakonia sacchari CGMCC 1.12102**	GCA_900100995.1	**Methylobacterium sp. CG08_land_8_20_14_0_20_71_15**	GCA_002778925.1
**Kosakonia sacchari SP1**	GCA_900109485.1	**Methylobacterium pseudosasicola BL36**	GCA_900114535.1
**Methylobacterium sp. 77**	GCA_000372825.1	**Methylobacterium sp. V23**	GCA_002917135.1
**Methylobacterium sp. 88A**	GCA_000376345.1	**Methylobacterium sp. UNCCL110**	GCA_000745415.1
**Methylobacterium sp. 10**	GCA_000519085.1	**Methylobacterium platani PMB02**	GCA_001653715.1
**Methylobacterium sp. WSM2598**	GCA_000379105.1	**Methylobacterium sp. ME121**	GCA_000974725.1
**Methylobacterium sp. EUR3 AL-11**	GCA_000526475.1	**Methylobacterium sp. TER-1**	GCA_004004555.1
**Methylobacterium radiotolerans ES_PA-B5**	GCA_004011495.1	**Methylobacterium radiotolerans NDB3P7**	GCA_001617335.1
**Methylobacterium sp. Leaf99**	GCA_001422375.1	**Methylobacterium radiotolerans RE1.2**	GCA_001981325.1
**Methylobacterium sp. DS2.3.33**	GCA_002928875.1	**Methylobacterium sp. 17J42-1**	GCA_004348265.1
**Methylobacterium sp. Leaf119**	GCA_001422935.1	**Methylobacterium radiotolerans 78c**	GCA_000878385.1
**Methylobacterium mesophilicum SR1.6/6**	GCA_000364445.1	**Methylobacterium sp.**	GCA_001724885.1
**Methylobacterium sp. MB200**	GCA_000333655.1	**Methylobacterium sp. CCH7-A2**	GCA_001557075.1
**Methylobacterium sp. Leaf93**	GCA_001422345.1	**Methylobacterium radiotolerans SB2**	GCA_001476225.1
**Methylobacterium sp. Leaf89**	GCA_001422215.1	**Methylobacterium radiotolerans SB3**	GCA_001477075.1
**Methylobacterium sp. Leaf121**	GCA_001423385.1	**Methylobacterium sp. L2-4**	GCA_000454305.1
**Methylobacterium sp. Leaf112**	GCA_001423295.1	**Methylobacterium indicum NS230**	GCA_001477085.1
**Methylobacterium sp. Leaf85**	GCA_001422125.1	**Methylobacterium sp. AWTP1-16**	GCA_003987715.1
**Methylobacterium sp. Leaf108**	GCA_001423265.1	**Methylobacterium sp. AWTP1-15**	GCA_003987805.1
**Methylobacterium sp. Leaf399**	GCA_001424525.1	**Methylobacterium indicum NS229**	GCA_001476215.1
**Methylobacterium sp. Leaf106**	GCA_001422895.1	**Methylobacterium sp. MIMD6**	GCA_003574465.1
**Methylobacterium sp. Leaf466**	GCA_001425565.1	**Methylobacterium tarhaniae DSM 25844**	GCA_001043955.1
**Methylobacterium sp. Leaf117**	GCA_001422985.1	**Methylobacterium indicum NS228**	GCA_001476615.1
**Methylobacterium sp. Leaf123**	GCA_001423405.1	**Methylobacterium indicum SE2.11**	GCA_001043895.1
**Methylobacterium sp. Leaf104**	GCA_001422885.1	**Methylobacterium aquaticum DSM 16371**	GCA_001043915.1
**Methylobacterium sp. Leaf102**	GCA_001422425.1	**Methylobacterium platani JCM 14648**	GCA_001043885.1

**Methylobacterium sp. Leaf122**	GCA_001423025.1	**Methylobacterium indicum SE3.6**	GCA_001043875.1
**Methylobacterium sp. Leaf456**	GCA_001425465.1	**Methylobacterium variabile DSM 16961**	GCA_001043975.1
**Methylobacterium sp. Leaf86**	GCA_001422165.1	**Belnapia moabensis DSM 16746**	GCA_000745835.1
**Methylobacterium sp. Leaf92**	GCA_001422305.1	**Belnapia sp. F-4-1**	GCA_000802185.1
**Methylobacterium sp. Leaf469**	GCA_001424745.1	**Belnapia rosea CGMCC 1.10758**	GCA_900104205.1
**Methylobacterium phyllostachyos BL47**	GCA_900103445.1		

Table S4. Genome sequencing of strains isolated from *A. salmiana*.

**Genome Name**	***Kosakonia sacchari* MJ18**	***Methylobacterium* sp. RAS18**	***Belnapia rosea* MJ22**
**IMG Genome ID**	2623620455	2823679482	2623620456
**NCBI Taxon ID**	1235834	2587127	938405
**Release Date**	21/08/2015	12/05/2019	21/08/2015
**Genome Size**	5538213	5855278	5994144
**Gene Count**	5351	5682	5795
**Scaffold Count**	36	37	119
**GC**	54	68	70
**Coding Base Count**	4920630	5003430	5397243
**CDS Count**	5194	5591	5726
**Compartment**	Root endosphere	Phyllosphere	Phyllosphere
**Reference**	Desgarennes, et al. 2014	This work	Desgarennes, et al. 2014

Table S5. Identification of phyllospheric bacteria.

**Name**	**Top-hit taxon**	**Top-hit strain**	**Similarity (%)**	**Taxonomy**
**AT32P**	*Leptolyngbya ohadii*	IS1	87.11	*Leptolyngbya* sp. AT32P
**AT21F**	*Nodosilinea epilithica*	Ru-6-11	98.66	*Nodosilinea* sp. AT21F
**RAS18**	*Methylobacterium extorquens*<br>*Methylobacterium pseudosasae*	IAM 12631<br>*BL44*	100.00	*Methylobacterium* sp. RAS18

References

Bankevich, A., Nurk, S., Antipov, D., Gurevich, A. A., Dvorkin, M., Kulikov, A. S., et al. (2012). SPAdes: a new genome assembly algorithm and its applications to single-cell sequencing. *J. Comput. Biol. J. Comput. Mol. Cell Biol.* 19, 455–477. doi:10.1089/cmb.2012.0021.

Desgarennes, D., Garrido, E., Torres-Gomez, M. J., Peña-Cabriales, J. J., and Partida-Martinez, L. P. (2014). Diazotrophic potential among bacterial communities associated with wild and cultivated Agave species. *FEMS Microbiol. Ecol.* 90, 844–857. doi:10.1111/1574-6941.12438.

Edgar, R. C. (2004). MUSCLE: Multiple sequence alignment with high accuracy and high throughput. *Nucleic Acids Research, 32*(5), 1792–1797. https://doi.org/10.1093/nar/gkh340

Huntemann, M., Ivanova, N. N., Mavromatis, K., Tripp, H. J., Paez-Espino, D., Tennessen, K., et al. (2016). The standard operating procedure of the DOE-JGI Metagenome Annotation Pipeline (MAP v.4). *Stand. Genomic Sci.* 11, 17. doi:10.1186/s40793-016-0138-x.

Ribeiro, F. J., Przybylski, D., Yin, S., Sharpe, T., Gnerre, S., Abouelleil, A., … Jaffe, D. B. (2012). Finished bacterial genomes from shotgun sequence data. *Genome Research, 22*(11), 2270–2277. https://doi.org/10.1101/gr.141515.112

Temraleeva, A. D., Dronova, S. A., Moskalenko, S. V., & Didovich, S. V. (2016). Modern methods for isolation, purification, and cultivation of soil cyanobacteria. *Microbiology, 85*(4), 389–399. https://doi.org/10.1134/S0026261716040159
